# *Reg4* deficiency aggravates pancreatitis by increasing mitochondrial cell death and fibrosis

**DOI:** 10.1038/s41419-024-06738-y

**Published:** 2024-05-20

**Authors:** Weihui Yan, Ying Wang, Ying Lu, Shicheng Peng, Bo Wu, Wei Cai, Yongtao Xiao

**Affiliations:** 1 Division of Pediatric Gastroenterology and Nutrition, Xin Hua Hospital, School of Medicine, Shanghai Jiao Tong University, Shanghai, 200092 China; 2 Shanghai Key Laboratory of Pediatric Gastroenterology and Nutrition, Shanghai, 200092 China; 3 Shanghai Institute for Pediatric Research, Shanghai, 200092 China; 4 Department of Pediatric Surgery, Xin Hua Hospital, School of Medicine, Shanghai Jiao Tong University, Shanghai, 200092 China

**Keywords:** Chronic pancreatitis, Chronic inflammation

## Abstract

Regenerating gene family member 4 (*Reg4*) has been implicated in acute pancreatitis, but its precise functions and involved mechanisms have remained unclear. Herein, we sought to investigate the contribution of *Reg4* to the pathogenesis of pancreatitis and evaluate its therapeutic effects in experimental pancreatitis. In acute pancreatitis, *Reg4* deletion increases inflammatory infiltrates and mitochondrial cell death and decreases autophagy recovery, which are rescued by the administration of recombinant *Reg4* (*rReg4*) protein. In chronic pancreatitis, *Reg4* deficiency aggravates inflammation and fibrosis and inhibits compensatory cell proliferation. Moreover, C-X-C motif ligand 12 (CXCL12)/C-X-C motif receptor 4 (CXCR4) axis is sustained and activated in *Reg4*-deficient pancreas. The detrimental effects of *Reg4* deletion are attenuated by the administration of the approved CXCR4 antagonist plerixafor (AMD3100). Mechanistically, *Reg4* mediates its function in pancreatitis potentially via binding its receptor exostosin-like glycosyltransferase 3 (*Extl3*). In conclusion, our findings suggest that *Reg4* exerts a therapeutic effect during pancreatitis by limiting inflammation and fibrosis and improving cellular regeneration.

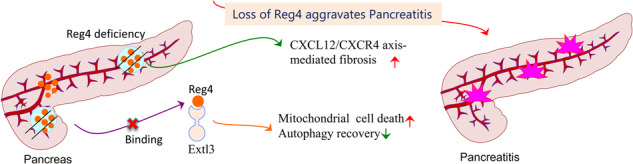

## Introduction

Regenerating gene family member 4 (*Reg4, REG4* in human) is the most recently discovered member of the regenerating (*Reg*) family [1, 2]. *REG4* gene is predominantly expressed in gastrointestinal tract tissues that include the pancreas [1, 3]. The aberrant expression of *REG4* in the pancreas has been linked to both pancreatitis and pancreatic cancer [4–8]. During acute pancreatitis (AP), the recombinant human *REG4* protein has been shown to protect against acinar cell necrosis [7, 8], but its precise roles and involved mechanisms in the pathogenesis of pancreatitis are remained poor understood. Herein, we knocked out *Reg4* in mice to test the hypothesis that loss of *Reg4* constitutes a pathogenic factor in pancreatitis. On the basis of this concept, we next used recombinant *Reg4* (*rReg4*) protein therapeutically to ameliorate caerulein-induced pancreatitis. Our results firstly showed that *Reg4* deletion resulted in the persistence of inflammatory and cell death in acute pancreatitis, and further confirmed *rReg4* could protect mice against cell death [8]. Moreover, *rReg4* also increased compensatory cell repaired via increasing the autophagy process. In chronic pancreatitis (CP), *Reg4* deletion aggravated pancreatic fibrosis and enhanced activation of the C-X-C motif ligand 12 (CXCL12)/C-X-C motif receptor 4 (CXCR4) axis. The aggravating effects of *Reg4* deletion were abrogated by the administration of the CXCR4 antagonist plerixafor (AMD3100). Taken together, our results suggest that *Reg4* has therapeutic effect for pancreatitis and deepened our understanding of the mechanisms underlying *Reg4* functions in pancreatitis.

## Results

### REG4 is reduced during the pancreatitis

**Fig. 1:**
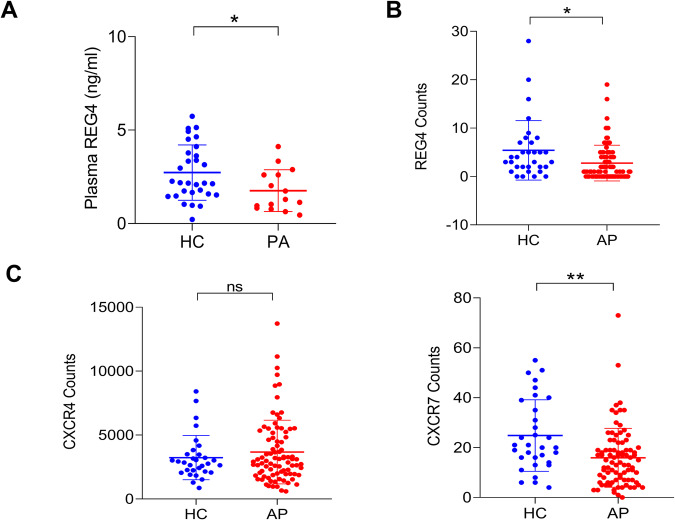
Blood REG4 is reduced during pancreatitis. **A** Enzyme-linked immunosorbent assay (ELISA) analysis of plasma REG4 in children with pancreatitis (*n* = 15) compared to the age-matched controls (*n* = 30). **B**, **C** Analysis of human REG4 mRNA, CXC chemokine receptor 4 (*CXCR4*) mRNA, and CXC chemokine receptor 7 (*CXCR7*) mRNA using publicly available datasets (dataset GSE194331, [11]). Acute pancreatitis (AP, *n* = 87), healthy controls (HCs, *n* = 32). All data are expressed as mean ± standard deviation (SD); unpaired, two-tailed Student’s *t*-test with or without Welch’s correction analysis for (**A**–**C**) (ns not significant; *P < 0.05, **P < 0.01).

In the human pancreas, single-cell transcriptomics analysis indicated that *REG4* mRNA was mainly expressed in exocrine glandular cells and ductal cells (https://proteinatlas.org [9, 10]). In mice pancreases, colorimetric in situ hybridization (CISH) analysis confirmed that *Reg4* mRNA was selectively expressed in glandular cells and ductal cells (Fig. S1A). Immunofluorescence (IF) staining showed that mouse REG4 protein was primarily located in islets and in goblet-like cell vesicles around the surfaces of ductal cells (Fig. S1B). The enzyme-linked immunosorbent assay (ELISA) analysis showed that the levels of blood REG4 protein were downregulated in children with pancreatitis (PA, *n* = 15) compared to age-matched controls (HC, *n* = 30, *P* < 0.05, Fig. 1A). Our analysis of publicly available datasets [11] (GSE194331) showed that human *REG4* mRNA expression was also decreased in the blood of patients with AP (*n* = 87) relative to healthy control (HC, *n* = 32, *P* < 0.05, Fig. 1B). We also observed that the CXC chemokine receptor 4 *(CXCR4*) mRNA increased in AP patients but not reach significant level (Fig. 1C). The CXC chemokine receptor 7 (*CXCR7*) mRNA was significantly reduced in AP patients (Fig. 1C). The ELISA assay depicted serum levels of mice REG4 were reduced during the periods of caerulein-induced pancreatitis (Fig. S2A). In addition, the REG4 proteins in mice pancreas were also reduced during pancreatitis (Fig. S2B, C).

### *Reg4* deficiency exacerbates experimental pancreatitis

**Fig. 2:**
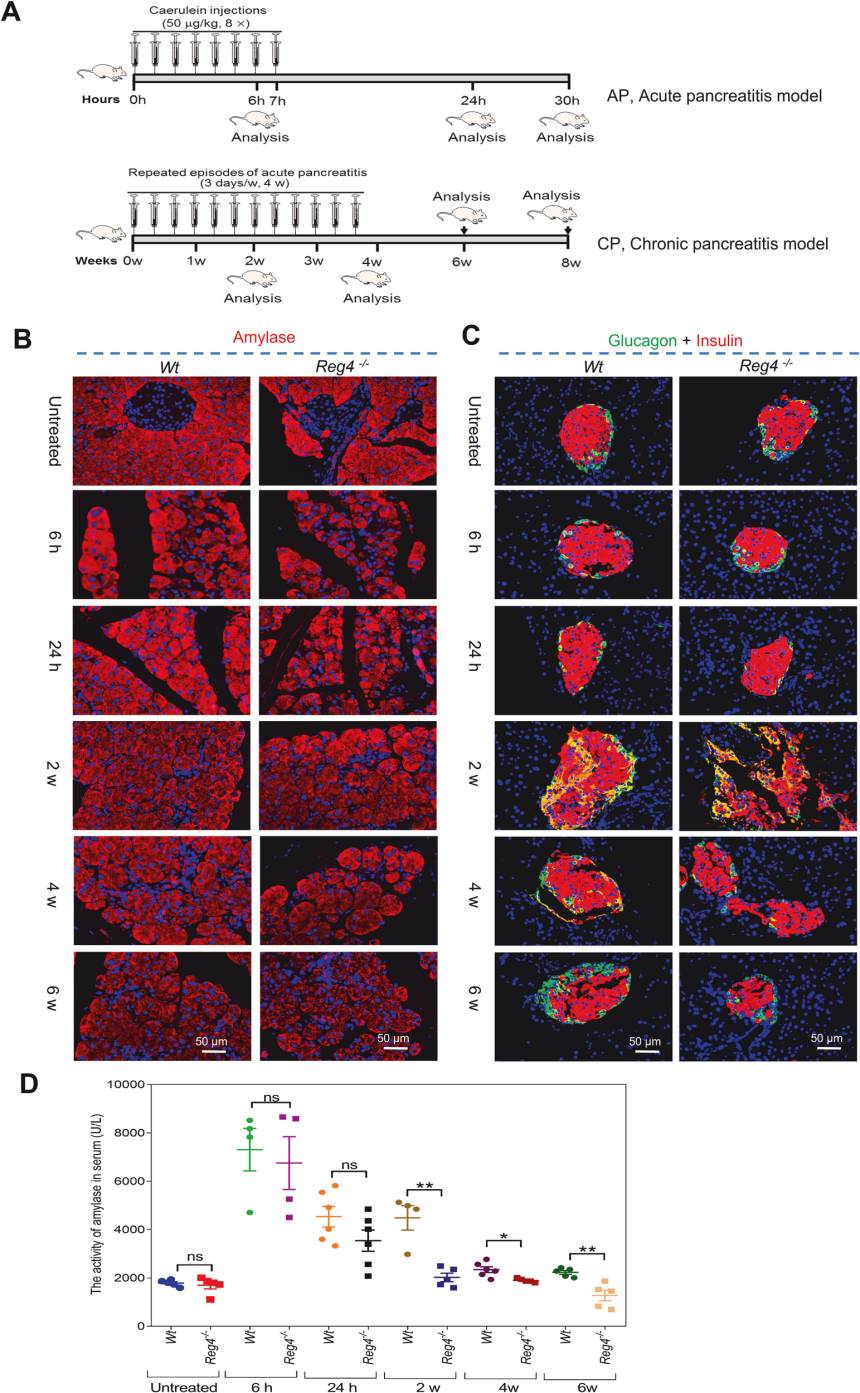
*Reg4* deficiency aggravates caerulein-induced pancreatic injuries. **A** Mice models of acute pancreatitis (AP) and chronic pancreatitis (CP). AP was induced in mice with 8 hourly injections of caerulein in *Reg4* knockout (*Reg4*^−^^*/*^^−^) mice and wild-type (*Wt*) mice. The animals were sacrificed for analysis 6, 24, or 30 h after the initial caerulein injection. CP was then established by repeated episodes of acute caerulein-induced pancreatitis over 4 weeks. Mice were killed 2, 4, 6 or 8 weeks after the first injection. **B**, **C** Immunofluorescence (IF) staining of pancreatic sections for Amylase (red) and glucagon (green) + insulin (red) during pancreatitis (for each group, *n* ≥ 4; *t* = 6 h, 24 h, 2 weeks, 4 weeks, or 6 weeks). **D** Serum activities of amylase in *Reg4*^−^^*/*^^−^ and *Wt* mice injected with caerulein were determined at the indicated time points (for each group, *n* ≥ 4; unpaired, two-tailed Student’s *t*-test with or without Welch’s correction analysis for (**D**); ns not significant, *P < 0.05, **P < 0.01).

To investigate the roles of Reg4 in pancreatitis, we initially depleted the *Reg4* in mice (*Reg4*^−^^*/*^^−^) and then established the models of pancreatitis as described previously (Fig. 2A) [12]. Our results showed that *Reg4*^−^^*/*^^−^ mice had a smaller and lighter pancreas than *Wt* mice (Fig. S3A, B). IF staining for acinar cells and islets showed that *Reg4*^−^^*/*^^−^ mice exhibited more pancreatic damage, with greater destruction of acinar and islet cells than that of *Wt* mice (Fig. 2B, C). Serum amylase activity peaked at approximately 6 h following the caerulein treatment, and we noted less activity in *Reg4*^−^^*/*^^−^ mice than in *Wt* mice (Fig. 2D). Histologically, hematoxylin and eosin (H&E)-stained sections of pancreas revealed a significant elevation in cellular necrosis in the caerulein-treated *Reg4*^−^^*/*^^−^ mice compared to the caerulein-treated *Wt* mice (Fig. S4A, B). We visualized immune cell infiltrates during caerulein-induced pancreatitis by staining for CD45 and observed more significant CD45 infiltration in *Reg4*^−^^*/*^^−^ mice at most time points relative to *Wt* mice during caerulein treatment (Fig. S4C, D). Surprisingly, F4/80^+^ macrophages were found to decrease in pancreases of *Reg4*^−^^*/*^^−^ mice compared to *Wt* mice (Fig. S5A–C).

### *Reg4* depletion reduces compensatory regeneration

**Fig. 3:**
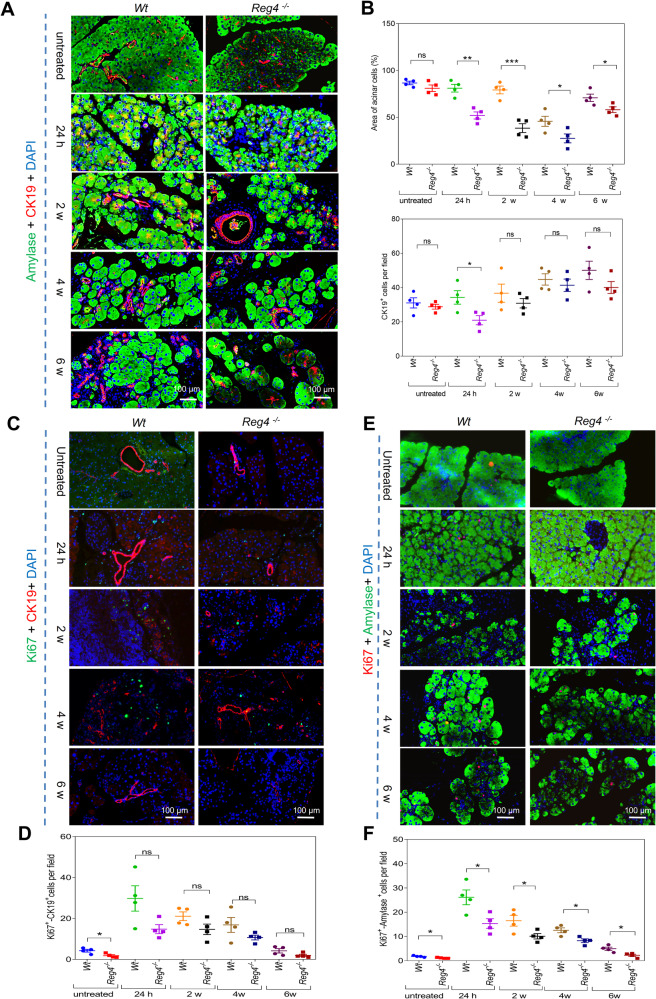
*Reg4* loss reduces regeneration of the ductal and acinar cells during pancreatitis. **A** IF staining of pancreatic sections for amylase (green) and CK19 (red) during pancreatitis (for each group, *n* = 4; *t* = 24 h, 2 weeks, 4 weeks, or 6 weeks). **B** Quantification of the acinar cell area and ductal cell number per field in (**A**). **C** IF staining of the pancreatic sections for Ki67 (green) and CK19 (red) during pancreatitis (for each group, *n* = 4; *t* = 24 h, 2 weeks, 4 weeks, or 6 weeks). **D** Quantification of the number of Ki67^+^-CK19^+^ cells per field in (**C**). **E** IF staining of the pancreatic sections for amylase (green) and Ki67 (red) during pancreatitis (*n* = 4 for each group; *t* = 24 h, 2 weeks, 4 weeks, or 6 weeks). **F** Quantification of the Ki67^+^-amylase^+^ cells per field in (**E**); unpaired, two-tailed Student’s *t*-test with or without Welch’s correction analysis for (**B**, **D**, and **F**) (ns not significant; *P < 0.05, **P < 0.01, ***P < 0.001).

**Fig. 4:**
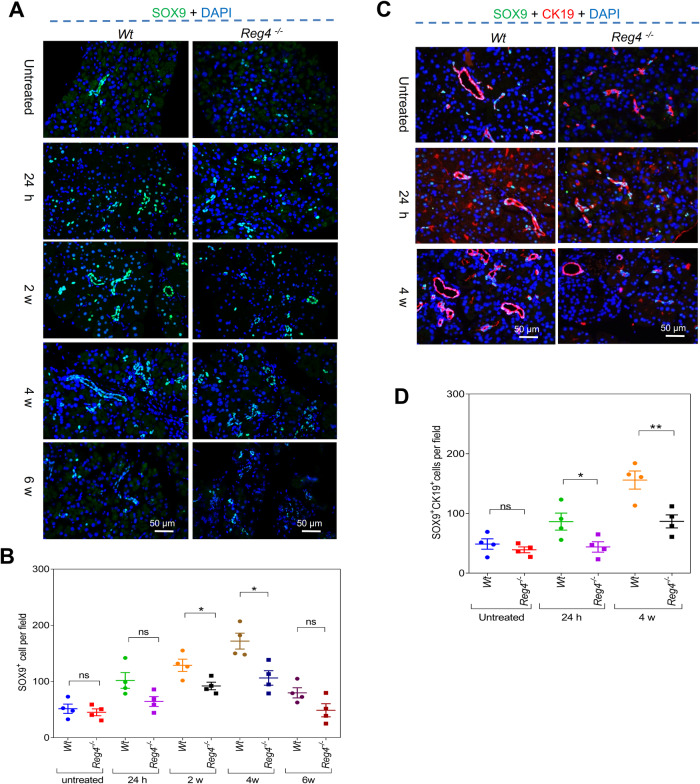
*Reg4* deficiency reduces SOX9-positive cells in the pancreas during pancreatitis. **A** IF staining of the pancreatic sections for SOX9 (green) during pancreatitis (*n* = 4 for each group; *t* = 24 h, 2 weeks, 4 weeks, or 6 weeks). **B** Quantification of the number of SOX9-positive cells per field in (**A**). **C** IF staining of the pancreatic sections for SOX9 (green) and CK19 (red) during pancreatitis (for each group, *n* = 4; *t* = 24 h or 4 weeks). **D** Quantification of the SOX9^+^-CK19^+^ cells per field in (**C**); unpaired, two-tailed Student’s *t-*test with or without Welch’s correction analysis for (**B** and **D**) (ns not significant; *P < 0.05, **P < 0.01).

IF staining for the acinar cell marker amylase and ductal cell marker cytokeratin 19 (CK19) showed that both the acinar cell area and the number of ductal cells were significantly reduced in the pancreases of *Reg4*^−^^*/*^^−^ mice compared to that of *Wt* mice during acute and chronic experimental pancreatitis (Fig. 3A, B). Immunohistochemical (IHC) staining of proliferating cell nuclear antigen (PCNA) indicated fewer proliferative cells in the pancreases of *Reg4*^−^^*/*^^−^ mice than in *Wt* mice, particularly during acute pancreatitis (Fig. S6A, B). Consistently, IF staining of antigen identified by proliferation marker protein antibody Ki67 also showed that the *Reg4*^−^^*/*^^−^ mice possessed fewer regenerative cells than did *Wt* mice (Fig. S7A, B). Moreover, the IF co-staining of Ki67 with CK19 or amylase indicated fewer regenerative acinar cells and ductal cells in *Reg4*^−^^*/*^^−^ mice than in *Wt* mice (Fig. 3C–F). The sex-determining region Y-box (SRY-box)-containing gene 9 (SOX9) is a marker of progenitor cells that give rise to endocrine and acinar cells [13, 14]. IF staining showed that SOX9^+^ cells were reduced in the pancreases of *Reg4*^−^^*/*^^−^ mice compared to *Wt* mice during pancreatitis (Fig. 4A, B). IF co-staining of SOX9 with CK19 indicated that SOX9 was principally expressed in CK19^+^ductal cells (Fig. 4C, D).

To examine whether therapeutic administration of *Reg4* reverses pancreatitis, recombinant *Reg4* (*rReg4*) was injected twice (at 0 h and 8 h) into *Reg4*^−^^*/*^^−^ mice with or without caerulein-introduction, and the mice were sacrificed 24 h after treatment (Fig. S8A). H&E-stained sections of the pancreas revealed a significant elevation in cellular necrosis in caerulein-treated *Reg4*^−^^*/*^^−^ mice that was counteracted by the administration of *rReg4* (Fig. S8B). Terminal deoxynucleotidyl transferase dUTP nick-end labeling (TUNEL) assays indicated that the increased percentage of TUNEL-positive cells (i.e., apoptotic cells) observed in the pancreases of caerulein-treated mice was attenuated by rReg4 treatment (Fig. S8C). Western-blot (WB) analysis indicated that *rReg4* administration induced mitochondrial protein B cell leukemia/lymphoma 2 (BCL-2), but inhibited the mitochondrial protein P53 upregulated modulator of apoptosis (PUMA), and then reduced the apoptotic mediators cleaved-caspase 3/9 in mouse pancreases during AP (Fig. S8D, E). In vitro, lactate dehydrogenase (LDH)-release analysis indicated that rReg4 administration inhibited caerulein- or arginine-induced necrosis in isolated acinar cells at the indicated time (Fig. S9A, B).

### Administration of *rReg4* ameliorates inflammation and autophagy recovery

**Fig. 5:**
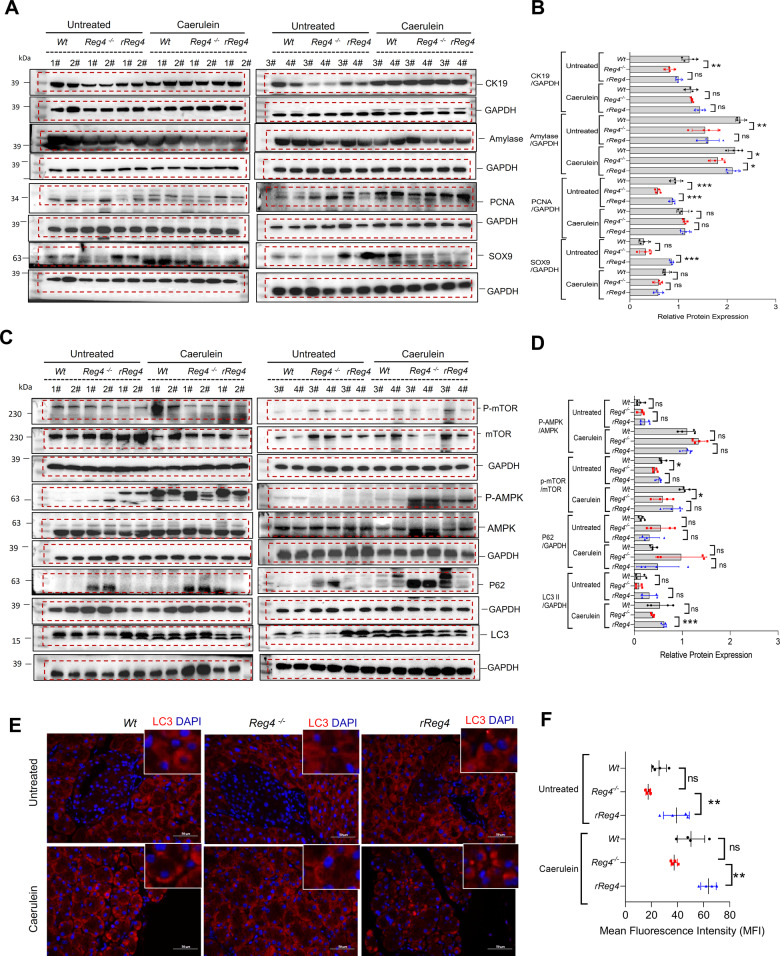
*rReg4* treatment increases cell regeneration and autophagy during pancreatitis. **A** Western-blot analysis for amylase, CK19, SOX9, and PCNA in the pancreas of *Wt*, *Reg4*^−^^*/*^^−^ mice, and *Reg4*^−^^*/*^^−^ mice with rReg4 treatment (*n* = 4 for each group, *t* = 24 h). **B** Quantification of (**A**). GAPDH was used as an internal reference. **C** Western-blot analysis for p-mTOR, mTOR, p-AMPK, AMPK, P62, and LC3 in the pancreas of *Wt*, *Reg4*^−^^*/*^^−^ mice, and *Reg4*^−^^*/*^^−^ mice with rReg4 treatment (*n* = 4 for each group, *t* = 24 h). **D** Quantification of the panel C. **E** Representative images of Immunofluorescence analysis for LC3 in the sections of the pancreas of *Wt*, *Reg4*^−^^*/*^^−^ and *Reg4*^−^^*/*^^−^ mice with rReg4 treatment mice. **F** Quantification of LC3 intensity in (**E**). Randomized one-way ANOVA for (**B**, **D**, and **F**) (ns not significant; *P < 0.05, **P < 0.01, ***P < 0.001).

IHC staining for CD45 or phosphorylated-STAT3 (p- STAT3) showed that *rReg4* treatment halted the immune cell infiltration and inflammatory responses during caerulein-induced pancreatitis (Fig. S10A, B). In addition, western-blot analysis indicated that proteins in the CXCL12 /CXCR4-signaling pathway increased significantly in the pancreases of *Reg4*^−^^*/*^^−^ mice but that their expression was attenuated by *rReg4* administration (Fig. S10C, D). WB analysis revealed that the protein levels of the ductal cell marker CK19, the acinar cell maker amylase, the proliferative cell marker PCNA, and the progenitor cell marker SOX9 decreased in the pancreases of *Reg4*^−^^*/*^^−^ mice with or without caerulein exposure, but their expression was improved by *rReg4* administration (Fig. 5A, B). The kinase mammalian target of rapamycin (mTOR) is a critical regulator of autophagy induction that can be negatively regulated by AMPK activation [15, 16]. WB analysis herein indicated that phosphorylated-AMPK (p-AMPK) increased and phosphorylated-mTOR (p-mTOR) decreased, with concomitantly reduced autophagy in the pancreases of *Reg4*^−^^*/*^^−^ mice during pancreatitis, but these alterations were circumvented by *rReg4* administration (Fig. 5C, D). We also observed that *Reg4* depletion reduced autophagy marker LC3 protein reduced and increased autophagy adaptor protein sequestosome 1 (SQSTM1/p62) in mice pancreas (Fig. 5C–F).

### *Reg4* deletion increases fibrosis via activating CXCL12/CXCR4 axis

**Fig. 6:**
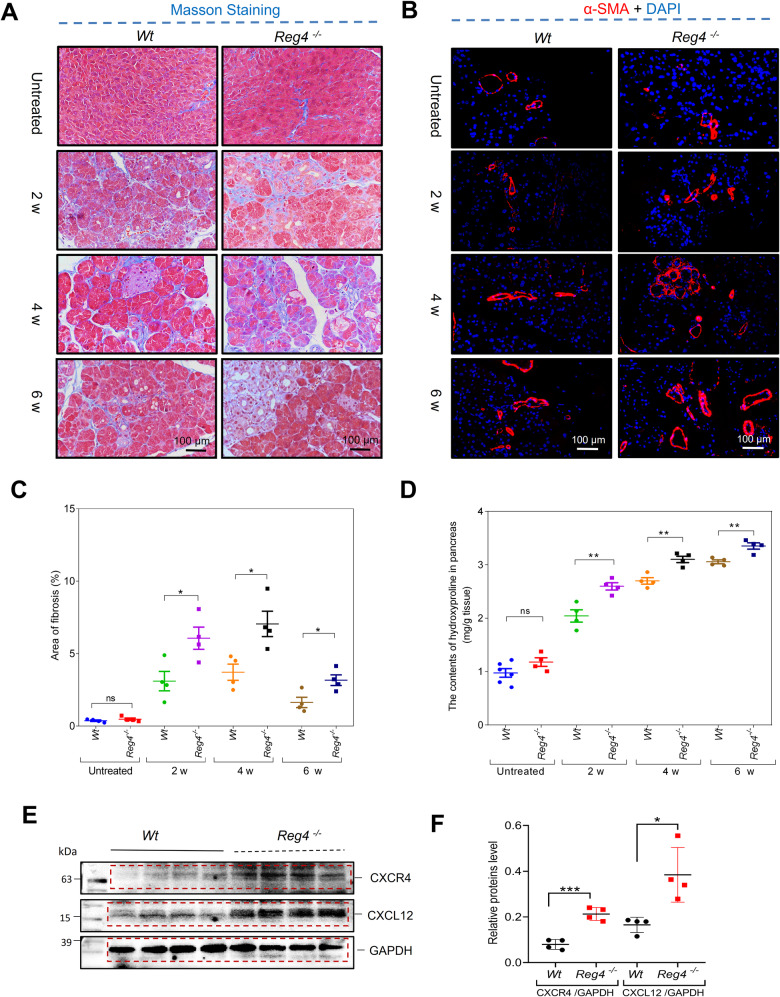
Ablation of *Reg4* leads to an increase in caerulein-induced fibrosis. **A**, **B** Representative images of Masson’s trichrome staining and immunofluorescence (IF) staining of α-SMA in the pancreas of *Wt* and *Reg4*^−^^*/*^^−^ mice (*n* = 4 for each group; *t* = 2, 4, or 6 weeks). **C** Quantification of the fibrotic area using Masson’s trichrome staining of images in (**A**). **D** Quantification of collagen content in the hydroxyproline assay in the pancreases of *Wt* and *Reg4*^−^^*/*^^−^ mice (*n* ≥ 4 for each group; *t* = 2, 4, or 6 weeks). **E** Western-blot analysis for CXCL12 and CXCR4 in the pancreases of *Wt* and *Reg4*^−^^*/*^^−^ mice after administration of caerulein for 1 week. **F** Quantification of panel E; GAPDH was used as the internal reference (*n* = 4); unpaired, two-tailed Student’s *t*-test with or without Welch’s correction analysis for **C**, **D**, and **F** (ns not significant; *P < 0.05, **P < 0.01, ***P < 0.001).

**Fig. 7:**
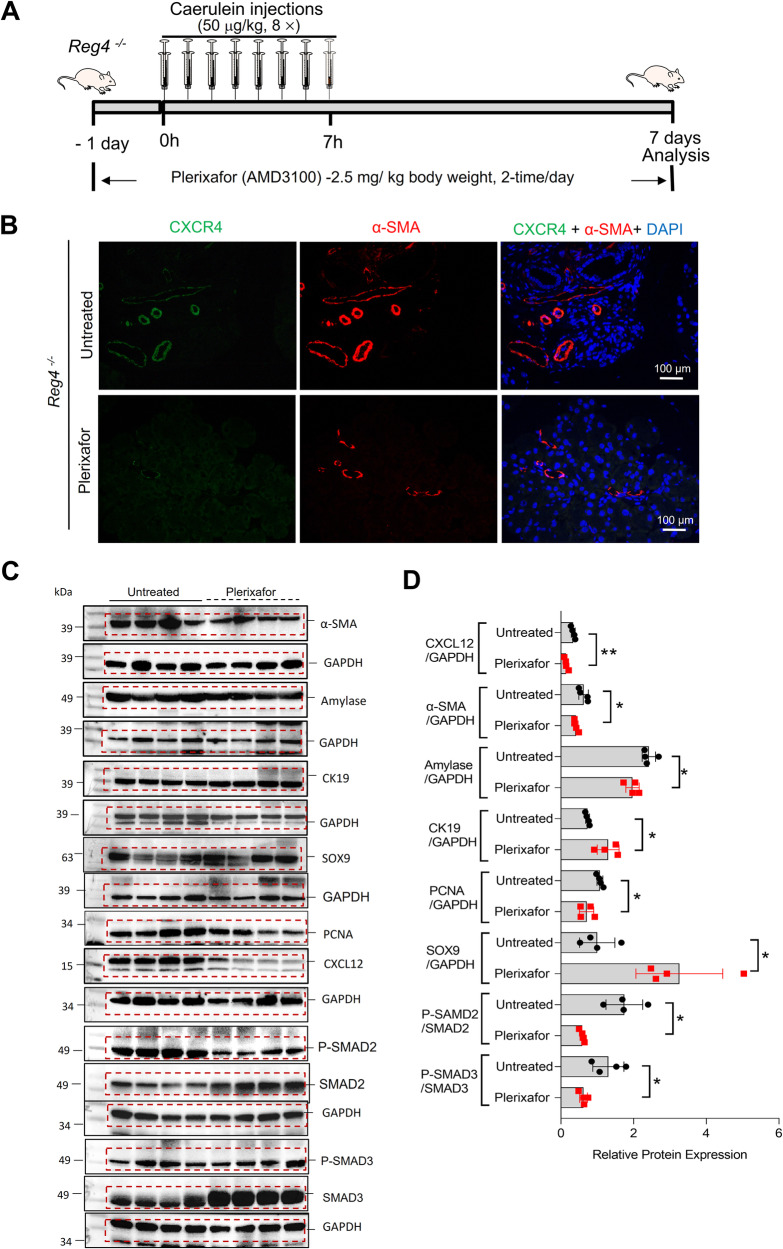
Inhibition of the CXCL12/CXCR4 axis attenuates caerulein-induced fibrosis in the pancreases of *Reg4-*deficient mice. **A** Treatment schedule of *Reg4*^−^^*/*^^−^ mice during acute caerulein-induced pancreatitis with or without the *CXCR4* antagonist plerixafor (AMD3100). Treatment with two daily injections (2.5 mg/kg body weight) initiated 1 day before induction of acute pancreatitis and continued until day 7 (*n* = 8 for each group). **B** IF staining of α-SMA and CXCR4 in the pancreatic sections of *Reg4*^−^^*/*^^−^ mice with or without plerixafor treatment (*n* ≥ 3 for each group). **C** Western-blot analysis for α-SMA, Amylase, CK19, SOX9, CXCL12, PCNA, phosphorylated SMAD2/3, SMAD2/3, and GAPDH in the pancreases of untreated mice or mice treated with plerixafor (*n* = 4). **D** Quantification of (**C**). GAPDH was used as the internal reference; unpaired, two-tailed Student’s *t-*test with or without Welch’s correction analysis for (**D**) (ns not significant; *P < 0.05, **P < 0.01).

Masson’s trichrome staining and IF staining of α-SMA both indicated that caerulein-induced significant pancreatic fibrosis in both *Wt* mice and *Reg4*^−^^*/*^^−^ mice but that it exerted a far greater impact on *Reg4*^−^^*/*^^−^ mice during CP (Fig. 6A–C). Indeed, we demonstrated that hydroxyproline levels were elevated in pancreases of *Reg4*^−^^*/*^^−^ mice compared with *Wt* mice following the caerulein treatment at the indicated time (2, 4, or 6 weeks; Fig. 6D). These results were consistent with our findings in AP, where the CXCL12/CXCR4 axis was robustly activated in CP samples of *Reg4*^−^^*/*^^−^ mice (Fig. 6E, F). A previous study indicated that CXCL12/CXCR4 axis activation contributed to the progression of fibrosis during experimental pancreatitis [12]. Accordingly, *Reg4* deficiency-induced fibrosis in the pancreas may be dependent upon the induction of the CXCL12/CXCR4 axis. To examine this, we treated the *Reg4*^−^^*/*^^−^ mice with the CXCR4 antagonist plerixafor (AMD3100) with two daily injections (2.5 mg/kg body weight) initiated 1 day before the induction of pancreatitis and which lasted until day 7 when the mice were sacrificed (Fig. 7A). Treatment of mice with plerixafor alleviated the features of fibrosis, commensurate with the declining expression of α-SMA and SMAD2/3 activation in the late phase of AP in *Reg4*^−^^*/*^^−^ mice (Fig. 7B–D). This CXCR4 antagonist also limited inflammation and mitigated atrophy by reducing CXCL12 and promoting the SOX9 and CK19 expression (Fig. 7B–D).

### EXTL3 acts as a putative receptor for REG4 in the pancreas

**Fig. 8:**
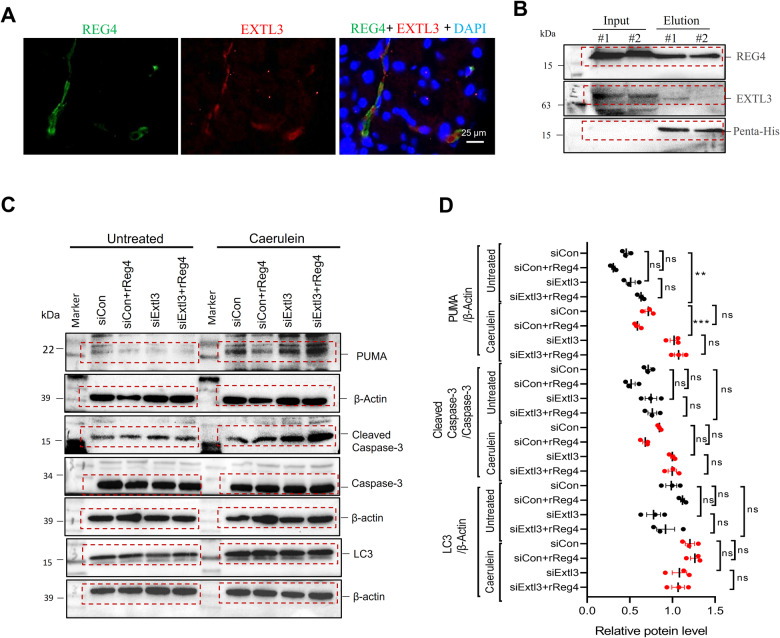
EXTL3 functions as a putative receptor for REG4 in the pancreas. **A** Co-localization of REG4 (green) with EXTL3 (red) in mice pancreas sections using immunofluorescence microscopy (*n* = 3). **B** Co-immunoprecipitation of REG4 and EXTL3 in mice pancreas (*n* = 2). **C** Knockdown of *Extl3* by siRNA (20 nM) in the context of the MIN6 cell line and treated with cerulean (200 μg/ml) for 6 h. Western-blot analysis for PUMA, Cleaved-Caspase 3, Caspase 3, LC3, and β-actin (*n* ≥ 3 for each treatment). **D** Quantification of (**C**). β-actin was used as the internal reference. Randomized one-way ANOVA for (**C**) (ns not significant; **P < 0.01).

Immunofluorescence (IF) analysis showed co-localization of EXTL3 (red) with REG4 (green) in the mice pancreas (Fig. 8A). The interaction between EXTL3 and REG4 in mice pancreas was further confirmed by the detection of EXTL3 by co-immunoprecipitation (Co-IP) with REG4 antibody (Fig. 8B). To confirm that EXTL3 is involved in regulating downstream REG4 protein, we examined the effect of silencing *Extl3* by siRNA in the MIN6 islet cell line. It showed that cerulean-induced cell apoptosis in MIN6 islet cell with increased the expression of cleaved-caspase 3 or PUMA, and these effects were attenuated by *rReg4* treatment (Fig. 8C, D). Silencing the *Extl3* aggravated cerulean-inducing cell death and in MIN6 islet cells (Fig. 8C, D). Moreover, *rReg4*-mediated pro-survival effects on the MIN6 islet cells was inhibited upon silencing *Extl3* (Fig. 8C, D).

## Discussion

Acute pancreatitis (AP), acute recurrent pancreatitis (ARP), and chronic pancreatitis (CP) have become significant health issues in the pediatric population [17–19]. Although recent studies have linked genetic and congenital abnormalities with pediatric ARP and CP [20–24], there are gaps in our knowledge of the natural history of pediatric pancreatitis and there are no established guidelines regarding treatment to improve clinical outcomes. Thus, there is an urgent need for novel therapies. We herein showed that *Reg4* constitutes a potentially novel therapeutic target in the treatment of pancreatitis by demonstrating a reduction in impaired regeneration and progressive fibrosis employing a mouse model. We first ascertained that both blood REG4 levels were reduced during pancreatitis in clinical samples. Second, *Reg4* deficiency exacerbated caerulein-induced experimental pancreatitis and impaired regeneration, and these effects were attenuated by the administration of recombinant *Reg4* protein. Finally, we noted that REG4 limited caerulein-induced fibrosis, at least in part, via inhibiting the activation of the CXCL12/CXCR4 pathway. EXTL3 may functions as the receptor for REG4 during pancreatitis.

AP is characterized by immune cell infiltration and a pronounced loss of acinar and islet cells. In this study, *Reg4* ablation increased immune cell infiltration and exacerbated the loss of acinar and islet cells, both of which were rescued by the administration of recombinant Reg4 protein. Mitochondrial dysfunction has been reported to play a central role in the development of AP [25]. Thus, Reg4 administration increased the levels of the anti-apoptotic mitochondrial molecule Bcl-2 and decreased Puma-mediated apoptosis, suggesting that Reg4 protected acinar and islet cells from cell death in pancreatitis by stabilizing mitochondria against death signals. Impaired mitochondrial function has been shown to lead to the accumulation of reactive oxygen species (ROS), endoplasmic reticulum (ER) stress, and autophagic dysfunction in mouse models of pancreatitis [26–28]. Consistent with these previous findings, we noted that *Reg4* deficiency impaired autophagic function by increasing P62 and decreasing LC3 levels during AP. AMPK is an important cellular energy sensor that regulates autophagy via the negative regulation of mTOR protein kinase [29–31]. AMPK can also inhibit mTORC1 signaling and thereby further suppress autophagosome maturation [30]. Intriguingly, *Reg4* deficiency leads to an increase in the activation of AMPK, suggesting that AMPK/mTOR signaling may be involved in *Reg4* deficiency-mediated autophagic inhibition during pancreatitis. During pancreatic regeneration, the loss of *Reg4* caused regenerative retardation with a decrease in proliferative cell number and SOX9 expression. SOX9 is notable as a source of progenitors for pancreatic growth and regeneration [32]. For example, pancreas-specific SOX9 depletion in mice resulted in severe pancreatic hypoplasia, suggesting that SOX9 functions in the expansion of the pool of multipotent progenitor cells that give rise to endocrine and acinar cells throughout development [13, 33]. SOX9 expression in pancreatic tissue sections and extracts was strongly induced after Reg4 protein administration in *Reg4* knockout mice, suggesting that *Reg4* contributes to regeneration by regulating SOX9. IF analyses suggested that SOX9-positive cells also stained positive for CK19, indicating that they were either duct cells or duct-like cells that originated from acinar cells due to acinar-ductal metaplasia. We herein also observed macrophage accumulation in the pancreas of *Reg4* knockout mice during regeneration. Since it was reported that depletion of macrophages impaired pancreatic regeneration [34], we hypothesize that *Reg4* may have contributed to the regeneration, in part, via attracting the macrophages. Intriguingly, we also demonstrated that *Reg4* deficiency increased pancreatic fibrosis via activation of the CXCL12/CXCR4 axis. Since the CXCL12/CXCR4 signaling has been identified as a mediator of fibrosis [12, 35], the pharmacological inhibition of CXCR4 in our study (limited inflammation and fibrosis) revealed the translational relevance of our model. Reg4 ablation may increase CXCL12/CXCR4 axis activation during pancreatitis and thereby potentially lead to stimulated TGF-β/SMADs profibrotic signaling, and this might then activate pancreatic stellate cells. Indeed, CXCR4 inhibition attenuated the phosphorylation of Smad2/3 during pancreatitis.

Exostosin tumor-like 3 (*EXTL3*) has been identified as a Reg receptor. *EXTL3* has been reported to act as the *Reg1α* receptor and involved in the regulation of pancreatic β-cells for maintaining the β-cell mass [36]. On other cell types, *Reg1α* stimulated neurite outgrowth via its receptor *EXTL3* [37]. Zhang et al., recently reported that *EXTL3* also functions as the receptor for *REG3B* in pancreatic ductal adenocarcinoma [38]. We here evaluated whether EXTL3 could interact with REG4. Dual immunofluorescence staining demonstrated co-localization of EXTL3 and REG4 in mice pancreas. Co-immunoprecipitation experiments confirmed an interaction between EXTL3 and REG4. Silencing *Extl3* in mouse acinar cells and islet cells resulted in a reduction of cell survival and with or without caerulein treatment. In addition, silencing *Extl3* abolished upon the effects mediated by *Reg4* protein administration on the acinar cells and islet cells. Silencing *Extl3* also reduced the activation of *Reg4-*mediated survival and regeneration signaling. We thus suggest *Extl3* may functions as the receptor for *Reg4* and mediates the activation of downstream signaling proteins during pancreatitis.

In conclusion, our study provides evidence for the protective function of *Reg4* in which this molecule attenuated inflammation, mitochondrial cell death, and fibrosis and promoted regeneration during pancreatitis. These findings emphasize the concept that *REG4* possesses potential therapeutic use in the prevention of pancreatitis and in the treatment of children with pancreatitis.

## Materials and methods

### Pediatric patients with pancreatitis

A total of 15 children with pancreatitis (seven children with acute pancreatitis, AP, and eight children with chronic pancreatitis, CP; age, 2–11 years, with eight boys and seven girls). Thirty age-matched control children (nine boys and 21 girls) from Xin Hua Hospital Affiliated to Shanghai Jiao Tong University School of Medicine, Shanghai, China participated in this study. Written consent was obtained from the parents of all participants. The study protocol was reviewed and approved by the Ethics Committee of Xin Hua Hospital, School of Medicine, Shanghai Jiao Tong University (XHEC-D-2022-268). Information on patients with pancreatitis is presented in Supplementary Table S1.

### *Reg4* knockout mice

*Reg4* knockout (*Reg4*^−^^*/*^^−^) mice were obtained from GemPharmatech (Nanjing, China). CRISPR/Cas9 technology was used to generate the *Reg4*^−^^*/*^^−^ mice by deleting the exon 3, and PCR was conducted to analyze mouse genotyping. The primers for wild-type (*Wt*) mice were 5′-CCAGTAAGAAACTGGAGCCTTCC-3′ and 5′- CTTACACTGCAATCACATTTCCTGC-3′. The primers for knockout mice were 5′- GTCCCACTGCTATGGGTACTTCC-3′ and 5′- ACCCTGGAGACCTGTTTCAGGTG −3′. The mice were housed under standard conditions, with water and standard chow provided throughout the study. All experiments were approved by the Ethics Committees of Xin Hua Hospital, School of Medicine, Shanghai Jiao Tong University (XHEC-F-2022-018). The main reagents used in this study are listed in Supplementary Table S2.

### Caerulein-induced experimental pancreatitis

Pancreatitis in mice (C57BL/6, about 6 weeks old) was induced by intraperitoneal injection of caerulein (a decapeptide analog of the potent pancreatic secretagogue cholecystokinin, CCK). For acute pancreatitis (AP), caerulein (50 μg/kg body weight) was administered to *Wt* mice and *Reg4*^−^^*/*^^−^ mice every hour for 8 consecutive hours and sacrificed at 0 h (*Wt* mice: female, *n* = 4, male, *n* = 4; *Reg4*^−^^*/*^^−^ mice: female, *n* = 4, male, *n* = 3), 6 h (*Wt* mice: female, *n* = 3, male, *n* = 3; *Reg4*^−^^*/*^^−^ mice: female, *n* = 3, male, *n* = 3), 24 h (*Wt* mice: female, *n* = 3, male, *n* = 3; *Reg4*^−^^*/*^^−^ mice: female, *n* = 3, male, *n* = 3), and 30 h (*Wt* mice: female, *n* = 3, male, *n* = 3; *Reg4*^−^^*/*^^−^ mice: female, *n* = 3, male, *n* = 3) after the first injection. For chronic pancreatitis (CP), *Wt* and *Reg4*^−^^*/*^^−^ mice were administered an intraperitoneal injection of caerulein (50 μg/kg, 8 hourly injections/day, repeated 3 days/week, and lasting for 4 weeks). The mice were then sacrificed at 2 weeks (*Wt* mice: female, *n* = 4, male, *n* = 4; *Reg4*^−^^*/*^^−^ mice: female, *n* = 4, male, *n* = 4), 4 weeks (*Wt* mice: female, *n* = 6, male, *n* = 6; *Reg4*^−^^*/*^^−^ mice: female, *n* = 5, male, *n* = 5), 6 weeks (*Wt* mice: female, *n* = 3, male, *n* = 3; *Reg4*^−^^*/*^^−^ mice: female, *n* = 3, male, *n* = 3), and 8 weeks (*Wt* mice: female, *n* = 3, male, *n* = 3; *Reg4*^−^^*/*^^−^ mice: female, *n* = 3, male, *n* = 3) after the first caerulein administration.

Caerulein (50 µg/kg body weight) was first administered in 8 hourly intraperitoneal injections, and to evaluate the role of *Reg4* protein in pancreatitis, recombinant mouse *Reg4* protein (*rReg4*, 500 μg/kg body weight) was injected intraperitoneally at 0 h and 8 h, and the mice were sacrificed 24 h (*Wt* mice: female, *n* = 8, male, *n* = 10; *Reg4*^−^^*/*^^−^ mice: female, *n* = 8, male, *n* = 9; *rReg4* treated *Reg4*^−^^*/*^^−^ mice: female, *n* = 8, male, *n* = 8) after the first injection (at 50 µg/kg body weight, 8 hourly intraperitoneal injections). To analyze the role of CXCL12/CXCR4 axis in pancreatic fibrosis, the mice were injected intraperitoneally with the CXCR4 inhibitor plerixafor (AMD3100) twice daily at a dosage of 2.5 mg/kg body weight on the day before the induction of AP and until the animals were killed after 7 days of the first caerulein administration (Untreated *Reg4*^−^^*/*^^−^ mice: female, *n* = 4, male, *n* = 4; AMD3100 treated *Reg4*^−^^*/*^^−^ mice: female, *n* = 4, male, *n* = 4). Serum was collected and centrifuged at 3500 × *g* for 20 min. The pancreatic samples were collected, snap-frozen in liquid nitrogen, and stored at −80 °C for further analysis, and other pancreatic samples were fixed in 4% paraformaldehyde (PFA) for morphological studies.

### Colorimetric in situ hybridization (CISH)

All process of the CISH were using the diethylpyrocarbonate (DEPC) water as described in a previous study [39]. Briefly, some pancreatic tissues from the mice were immediately fixed in 4% paraformaldehyde (PFA) and embedded in paraffin, and then were deparaffinized in xylene and rehydrated in serial dilutions of ethanol. The slides were incubated with proteinase K (20 μg/mL) at 37 °C for 30 min and then washed with phosphate-buffered saline (PBS, three times for 5 min each) and hybridized overnight with a probe specific to Reg4 at a concentration of 8 μg/mL in a moist chamber at 37 °C. The probe sequence for mouse *Reg4* was 5′-DIG-GCACAGGAAGTGTTGGCGGTTGG-3′, and this *Reg4* probe was labeled with digoxin (DIG) at the 5′ end.

### Hydroxyproline determination in the pancreas

A Hydroxyproline Assay Kit (MAK008, Sigma) was used following the manufacturer’s instructions. Briefly, approximately 10 mg of pancreas tissues were used to determine hydroxyproline content. Tissues were homogenized in 100 μl of water with 100 μl of concentrated hydrochloric acid and hydrolyzed at 120 °C for 3 h. A chloramine T/oxidation buffer mixture was added into the homogenates and cultured for 5 min at room temperature (RT), and tissue culture continued with 4-dimethylaminobenzaldehyde (DMAB) reagent at 60 °C for 90 min. The optical absorbance was measured at 560 nm, and the hydroxyproline content was expressed as mg per gram of pancreas.

### Biochemical measurements and Enzyme-linked immunosorbent assay (ELISA)

The levels of amylase in the serum were quantified using a kit (Amylase kit, C016-1-1, Nanjing Jiancheng Bioengineering Institute, Nanjing, China) according to the manufacturer’s instructions. The concentrations of Reg4 in the serum of mice were determined using an ELISA kit (NBP2-82151, Novus) according to the instructions of the manufacturer. To determine the levels of REG4 in clinical samples, we used the human REG4 ELISA Kit (Shxybio, Shanghai, China) according to the manufacturer’s instructions.

### Histology

Sections were mounted on positively charged slides after cutting at a 4-μm thickness, baked at 65 °C for 1 h, and then stored at RT for later use. The sections were then stained with Haematoxylin and Eosin (H&E) staining for histological analysis. Necrosis was determined in at least six fields of view/section using CaseViewer, with each group having over six sample sections. Fibrosis was assessed using Masson’s trichrome stain according to the manufacturer’s instructions (Servicebio, Wuhan, China). Collagen fibers were stained blue, the nuclei were stained black, and the background was stained red. Fibrosis was quantified by determining the blue areas of at least six fields of view/section with CaseViewer. Each group contained over five sections.

### Immunohistochemical (IHC) and Immunofluorescence (IF) staining

Briefly, the sections were mounted on positively charged slides after cutting at 4-µm thickness, baked at 65 °C for 1 h, and incubated with xylol and descending concentrations of ethanol. Endogenous peroxidases were blocked using 0.3% H_2_O_2_ for 10 min at RT, and after antigen retrieval, non-specific binding was blocked using 5% bovine serum albumin for 30 min at RT. The antibodies were applied at their optimal concentrations overnight in a wet chamber at 4 °C. The slides were then rinsed with PBS and incubated with the appropriate secondary antibody for 1 h at RT. The slides were rinsed with PBS and counterstained with hematoxylin or 2-(4-amidinophenyl)-6-indolecarbamidine dihydrochloride (DAPI). Morphometric assessments were conducted in 10 randomly chosen microscopic high-power fields (hpf) per sample (*n* = 3–6 for each group). Images were analyzed with Image-Pro Plus software (Media Cybernetics, Rockville, MD, USA). Information regarding antibodies is presented in Supplementary Table S3.

### TdT-mediated dUTP nick-end labeling (TUNEL) analysis

A TUNEL kit (G1507, Servicebio, Wuhan, China) was used to analyze cell death or the numbers of apoptotic cells in the pancreatic sections according to the manufacturer’s instructions. Positive cells were visualized using diaminobenzidine (DAB) chromogen or fluorescence staining. The images were quantified using at least six fields of view/section with CaseViewer (each group contained more than three sample sections).

### Isolation of pancreatic acinar cells

Pancreatic tissues were removed carefully from adult mice and suspended in Waymouth’s medium. Tissues were then cut into small pieces (1–3 mm^3^) and digested in 10 mL of Hank’s Balanced Salt Solution (HBSS) containing 10 mM N-2-hydroxyethylpiperazine-N-2-ethane sulfonic acid (HEPES), 200 U/ml collagenase IA, and 0.25 mg/ml trypsin inhibitor at 37 °C for 15–20 min. Thereafter, cells were collected by centrifugation at 450 × *g* at 4 °C for 3 min, and the cellular pellets were suspended in 7 ml of Waymouth’s medium (containing 2.5% FBS, 1% penicillin-streptomycin mixture, and 0.25 mg/ml trypsin inhibitor). Digested cells were filtered with a 100 μm cell strainer, and isolated acinar cells were seeded in a six-well culture dish and cultured at 37 °C in an atmosphere of 5% (v/v) CO_2_ at high humidity. For treatments, 200 μg/l caerulein or 2 mg/ml arginine with or without 20 μg/ml *rReg4* was added into the culture medium for 0, 2, 4, or 8 h.

### Co-immunoprecipitation (Co-IP) assays

Total 30 mg protein lysates from mice pancreas was incubated with rReg4 protein (100 µg/ml) at 4 °C overnight to form the REG4/EXTL3 complexes. Binding was assessed by Ni-NTA-agarose immunoprecipitation followed by elution. The binding between REG4 and EXTL3 was analyzed by Western blotting with anti-EXTL3, anti-REG4, and anti-His antibodies. An aliquot of supernatant was kept as the input lysate prior to Immunoprecipitation (IP).

### Cell culture and treatment

The mouse pancreatic islet cells MIN6 were obtained from the Wuhan Servicebio Technology Co., Ltd. This cell line was authenticated by its manufacturers and was test by mycoplasma contamination before using. MIN6 cells were cultured in a 37 °C and 5% CO_2_ incubator. MIN6 cells were maintained in RPMI1640 medium (Invitrogen) with supplemented with 10% fetal bovine serum, 100 µg/mL penicillin, and 100 µg/mL streptomycin. Small interfering RNA (siRNA) oligonucleotides targeting Extl3 (#SR421199) were purchased from the Origene. MIN6 cells were transfected with siRNA (20 nM) or scrambled siRNA according to the manufacturer’s instructions using lipofectamine™ RNAiMAX (#13778, Thermo Fisher). After 72 h of transfection, the cells were treated with 20 μg/ml *rReg4* protein, 200 μg/l cerulean or combination of them for 6 h. The treated cells were next to performed Western-blot analysis.

### Lactate dehydrogenase (LDH) cytotoxicity assay

Cytotoxicity was measured using an LDH Cytotoxicity Assay Kit (CK12, Dojindo, Japan) according to the manufacturer’s instructions. Briefly, adding 10 μl of 10 × lysis buffer to the blank column and incubated the plate at 37 °C for 45 min with the appropriate level of CO_2_. Transferring 50 μl of the supernatant containing the treated acinar cells to a 96-well flat-bottom plate and added 50 µl of the reaction mixture to each sample well. The plate was incubated at RT for 30 min, protected from light. Finally, adding 50 μl of stop solution to each sample well, and measured the optical absorbance at both 490 nm and 680 nm.

### Protein extraction and Western-blot analysis

Briefly, approximately 50 mg of pancreatic tissues or isolated acinar cells was homogenized in RIPA buffer supplemented with a protease inhibitor cocktail (G2006, Servicebio, China) and trypsin inhibitor (XY210088, X-Y Biotechnology, China). We then used a Bicinchoninic Acid (BCA) Protein Assay Kit (#23225, Pierce) to determine the protein concentrations. Equal amounts of protein were separated on 10% Bis-Tris gels and transferred onto polyvinylidene difluoride (PVDF) membranes. The membranes were then incubated with the primary antibodies overnight at 4 °C after blocking with 5% non-fat milk at RT for 60 min. The membranes were washed three times for 30 min with Tris-Buffered SalineTween20 (TBST), containing 0.1% Tween-20, and then incubated with secondary antibodies. After the final washes with TBST, the signals were detected using Pierce^TM^ Enhanced Chemiluminescence (ECL) Western Blotting Substrate (#32106, Thermo Fisher). The respective antibodies are listed in Supplementary Table S3.

### Statistical analysis

All data are expressed as mean ± SD and analyzed by GraphPad Prism 8 Software (GraphPad Software, San Diego, CA). For comparisons between two groups, we determined statistical significance with the two-tailed, unpaired Student’s *t*-test (for parametric data) and Mann–Whitney *U*-test (for data that did not follow a normal distribution). For multiple-group comparisons, a one-way ANOVA with Tukey’s post-hoc test (for parametric data) and Kruskal–Wallis with Dunn’s test (nonparametric) were adopted. A *P*-value less than 0.05 was considered to be statistically significant.

### Supplementary information

Supplementary Tables and Figures

Original WB bands

## Data Availability

The data that support the findings of this study are available from the corresponding authors.
