# Supplementary material for: Reg4 deficiency aggravates pancreatitis by increasing mitochondrial cell death and fibrosis
Source: Cell Death Dis. 2024 May 20;15(5):348. doi: 10.1038/s41419-024-06738-y (PMC11106239; doi:10.1038/s41419-024-06738-y)
Supplement: Supplementary file 1 — Supplementary Tables and Figures [file 41419_2024_6738_MOESM1_ESM.docx]

# Supplementary Materials

***Reg4* deficiency aggravates pancreatitis by increasing mitochondrial cell death and fibrosis**

Weihui Yan, Ying Wang, Ying Lu, [Shicheng](https://www.ncbi.nlm.nih.gov/pubmed/?term=Chen%20S%5BAuthor%5D&cauthor=true&cauthor_uid=34168124) Peng, Bo Wu, Wei Cai and Yongtao Xiao

**Contents**

Supplementary Tables 1 - 3 Pages 2 - 6

Supplementary Figures 1 - 7 Pages 7 - 23

**Supplementary Tables**

**Table S1. Information of children with pancreatitis and controls.**

| **NO** | **Gender** | **Years** | **Diseases** | **Amlyase (IU/L)** | **Lipase (U/L)** |
| --- | --- | --- | --- | --- | --- |
| 1 | Female | 3 | acute pancreatitis | 51 | 473 |
| 2 | Female | 4 | acute pancreatitis | 393 | 498 |
| 3 | Female | 2 | acute pancreatitis | 344 | 380 |
| 4 | Female | 6 | chronic pancreatitis | 474 | 3000 |
| 5 | Male | 11 | acute pancreatitis | 346 | 461 |
| 6 | Female | 4 | acute pancreatitis | 392 | 543 |
| 7 | Male | 5 | chronic pancreatitis | 158 | 622 |
| 8 | Male | 6 | chronic pancreatitis | 399 | 630 |
| 9 | Male | 9 | acute pancreatitis | 340 | 317 |
| 10 | Male | 4 | chronic pancreatitis | 427 | 725 |
| 11 | Male | 10 | chronic pancreatitis | 120 | 284 |
| 12 | Female | 4 | chronic pancreatitis | 260 | 611 |
| 13 | Male | 9 | chronic pancreatitis | 116 | 161 |
| 14 | Male | 8 | chronic pancreatitis | 474 | 266 |
| 15 | Female | 6 | acute pancreatitis | 183 | 192 |
| 16 | Female | 9 | prepubertal development |  |  |
| 17 | Female | 8 | premature breast development |  |  |
| 18 | Male | 10 | prepubertal development |  |  |
| 19 | Female | 8 | premature breast development |  |  |
| 20 | Female | 9 | premature breast development |  |  |
| 21 | Female | 9 | sexual precocity |  |  |
| 22 | Male | 10 | children hyperactivity |  |  |
| 23 | Female | 8 | premature breast development |  |  |
| 24 | Female | 9 | premature breast development |  |  |
| 25 | Male | 8 | children hyperactivity |  |  |
| 26 | Male | 9 | children hyperactivity |  |  |
| 27 | Male | 12 | prepubertal development |  |  |
| 28 | Female | 8 | premature breast development |  |  |
| 29 | Female | 8 | premature breast development |  |  |
| 30 | Female | 9 | premature breast development |  |  |
| 31 | Female | 8 | premature breast development |  |  |
| 32 | Male | 8 | children hyperactivity |  |  |
| 33 | Male | 10 | malnutrition |  |  |
| 34 | Female | 8 | premature breast development |  |  |
| 35 | Female | 9 | premature breast development |  |  |
| 36 | Male | 7 | children hyperactivity |  |  |
| 37 | Female | 9 | premature breast development |  |  |
| 38 | Female | 11 | prepubertal development |  |  |
| 39 | Female | 10 | sexual precocity |  |  |
| 40 | Female | 11 | premature breast development |  |  |
| 41 | Male | 12 | prepubertal development |  |  |
| 42 | Female | 8 | sexual precocity |  |  |
| 43 | Female | 9 | prepubertal development |  |  |
| 44 | Female | 12 | prepubertal development |  |  |
| 45 | Female | 10 | prepubertal development |  |  |

**Table S2.** **Main reagents and Source**

| **Reagent** | **Catalog#** | **Source** |
| --- | --- | --- |
| HBSS (Ca2+/Mg2+) | G4204-500mL | Servicebio |
| HBSS (without Ca2+/Mg2+) | G4203-500mL | Servicebio |
| ceruletide | HY-A0190 | Medchemexpress |
| CXCR4 inhibitor Plerixafor(AMD3100) | HY-10046 | Medchemexpress |
| Mouse Direct PCR Kit | B40013 | bimake |
| 2x M-PCR OPTI™ Mix (Dye Plus) | B45012 | bimake |
| YeaRed Nucleic Acid Gel Stain | 10202ES76 | YEASEN |
| RIPA buffer | G2002-100ML | Servicebio |
| protease inhibitor cocktail | G2006 | Servicebio |
| trypsin inhibitor | XY210088 | X-Y Biotechnology |
| Bicinchoninic Acid (BCA) Protein Assay Kit | 23225 | Pierce |
| chemiluminescence (ECL) Western Blotting Substrate | 32106 | Thermo Fisher |
| Tween20 | 0777-1L | VWR Life Science |
| Protein Free Rapid Blocking Buffer | PS108P | EpiZyme |
| SDS-PAGE Transfer Buffer power | G2017 | Servicebio |
| Tris Buffered Saline powder | G0001 | Servicebio |
| Tris-MOPS-SDS Running Buffer Powder | M00138 | GenScript |
| Trypsin | G4001-100ML | Servicebio |
| L-Arginine | A5006-100G | SIGMA |
| LDH Cytotoxicity Assay Kit | C20301 | invitrogen |
| α-Amylase Assay Kit | C016-1-1 | Nanjing Jiancheng Bioengineering Institute |
| Lipofectamine™ RNAiMAX | 13778 | ThermoFisher |

**Table S3. Antibody information**

| **Antibody** | **Source** | **Catalog#** | **Application/dilution** |
| --- | --- | --- | --- |
| REG4 | ABCAM | Ab204171 | IF(1:150) |
| EXTL3 | Proteintech | 10588-1-AP | WB (1:1000)/ IF(1:200), IP |
| REG4 | ABCAM | ab256818 | WB (1:1500), IP |
| Amylase | Santa Cruz Biotechnology | SC-46657 | IF(1:100)/WB (1:20000) |
| Insulin | Servicebio | GB11334 | IF(1:150-1:1000) |
| CD45 | Servicebio | GB11066 | IHC(1:500)/IF(1:1000) |
| Glucogon | Servicebio | GB113496 | IF(1:500-1:1000) |
| PCNA | Servicebio | GB11010-1 | IHC(1:2000) |
| Ki67 | Servicebio | GB111141 | IF(1:50) |
| α-SMA | Servicebio | GB111364 | IHC(1:1000)/IF(1:500) |
| F4/80 | Servicebio | GB11027 | IHC(1:1000)/IF(1：500) |
| Cleaved-Caspase3 | Servicebio | GB11532 | IHC(1:500) |
| CXCL12 | Servicebio | GB11624 | IF(1:300)/WB (1:1000) |
| CK19 | Servicebio | GB11197 | WB (1:1000)/ IF(1:500) |
| CXCR4 | invitrogen | 14-9991-82 | WB (1:1000)/ IF(1:100) |
| Phospho-STAT3 | Cell Signaling Technology | 9145S | IHC(1:200) |
| PCNA | BIOSS | bsm-33035M | WB (1:1000) |
| Microtubule-associated protein 1, light chain 3/LC3 | Cell Signaling Technology ,CST | #12741S | WB (1:1000); IF (1:200) |
| Phospho-AMPK | Cell Signaling Technology | 2535S | WB (1:1000) |
| AMPK | Cell Signaling Technology | 2532S | WB (1:1000) |
| Phospho-SMAD3 | Cell Signaling Technology | 9520P | WB (1:1000) |
| SMAD3 | Cell Signaling Technology | 9523P | WB (1:1000) |
| Phospho-SMAD2 | Cell Signaling Technology | 3108P | WB (1:1000) |
| SMAD2 | Cell Signaling Technology | 5339P | WB (1:1000) |
| BCL2 | BBI Life Sciences | D260117-0025 | WB (1:1000) |
| SOX9 | NOVUS | NBP2-67690 | WB (1:1000)/ IF(1:300) |
| Phospho-mTOR （S2448） | Cell Signaling Technology | 2976P | WB (1:1000) |
| mTOR | Cell Signaling Technology | 2983P | WB (1:1000) |
| PUMA | Cell Signaling Technology | 12450S | WB (1:1000) |
| GAPDH | Cell Signaling Technology | 5174S | WB (1:1000) |
| P62 (SQSTM1) | Cell Signaling Technology | 23214S | WB (1:1000) |
| Cleaved Caspase-3 | Cell Signaling Technology | 9664T | WB (1:1000) |
| Caspase-3 | Cell Signaling Technology | 9665P | WB (1:1000) |
| α-SMA | ABCAM | Ab32575 | WB (1:1000) |
| Cleaved Caspase-9 | Cell Signaling Technology | 9501P | WB (1:1000) |
| Caspase-9 | Cell Signaling Technology | 9504S | WB (1:1000) |
| His antibody(BSA free) | QIAGEN | 34660 | IP |

**Supplementary Figures**

**Figure S1 The Reg4 expression in mice pancreas. A** The *colorimetric in situ hybridization (CISH)* analysis for *Reg4* mRNA in mice pancreas. Arrows indicated positive signals. n ≥3. **B** Immunofluorescence (IF) staining of mouse REG4 in combination with the acinar cell-marker amylase, the islet maker insulin, or the ductal cell-marker cytokeratin19 (CK19) in pancreas.

**
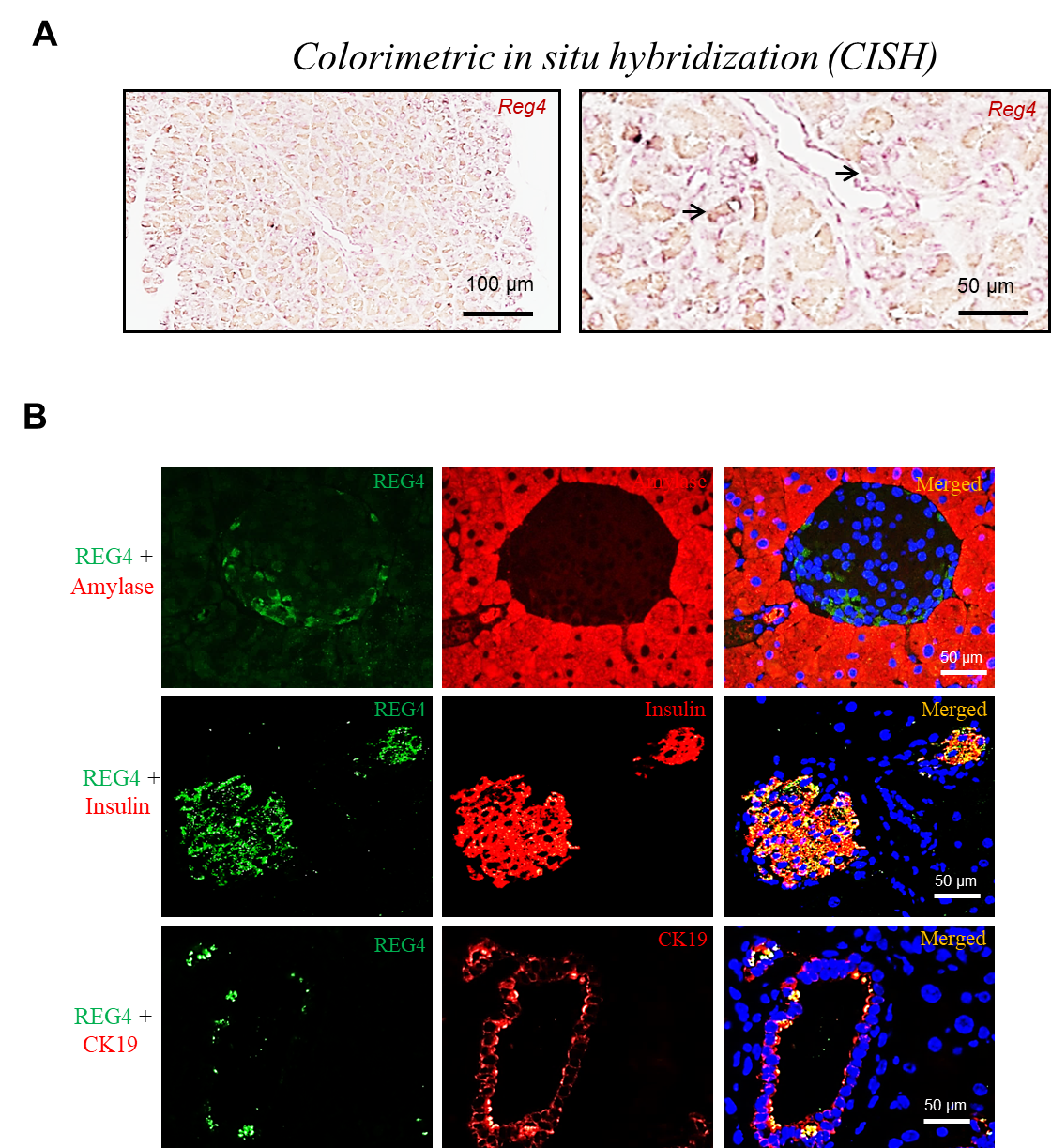
**

**Figure S2 Reg4 reduces during the experimental pancreatitis. A** enzyme linked immunosorbent assay (ELISA) analysis for the serum Reg4 during the mice pancreatitis. **B** Western-blot analysis for REG4 and GAPDH in the pancreases of *Wt*, mice with Caerulein treatment (n = 4 for each group). **C** Quantification of panel B; GAPDH was used as the internal reference. Randomized one-way ANOVA was used.for panel **A** and **C**. (ns, not significant; ***P* < 0.01, ****P* < 0.001).

**
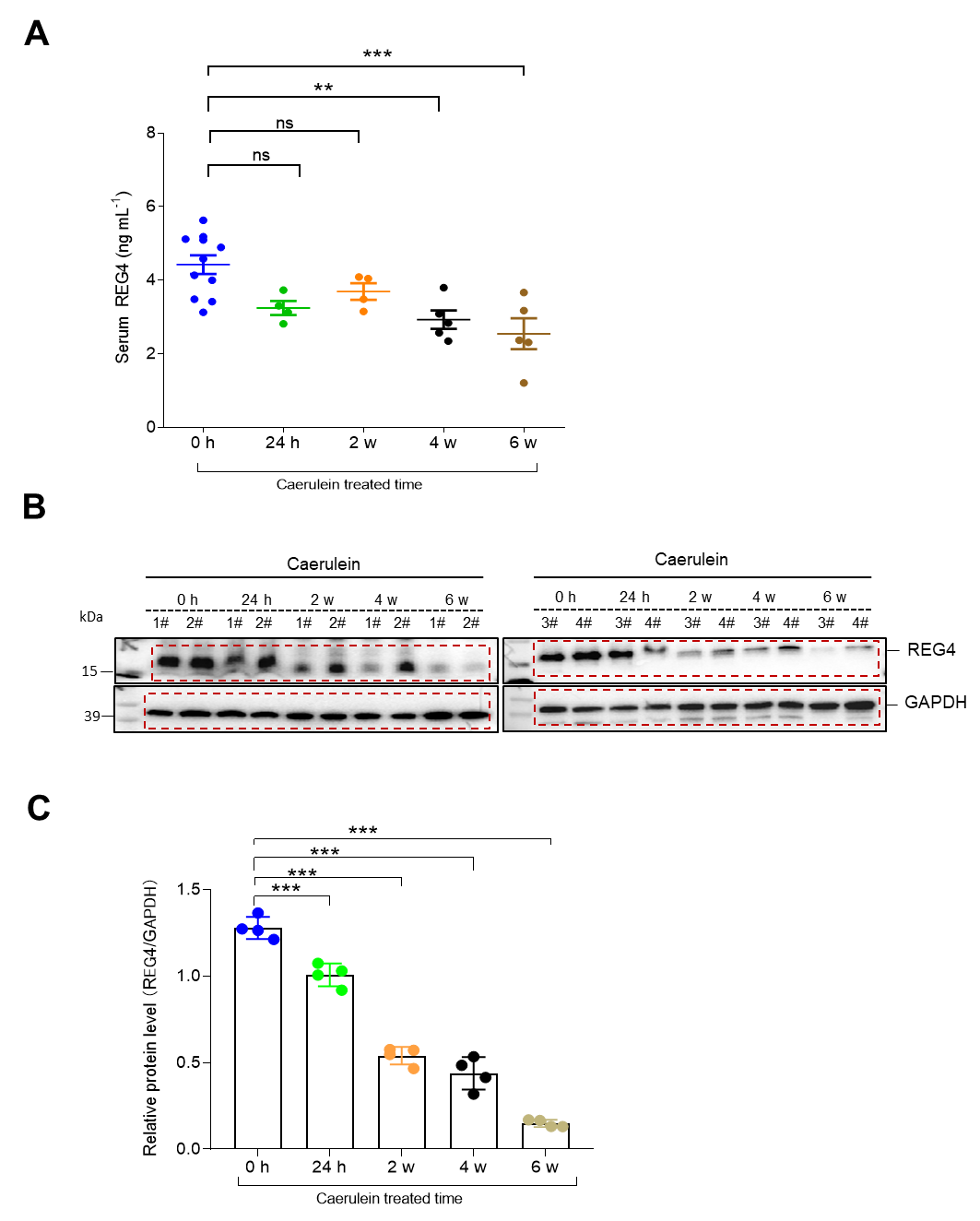
**

**Figure S3 Loss of Reg4 decreases the growth of pancreas. A** Representative images of pancreas from the *Wt* and *Reg4^-/-^* mice. **B** The weight of pancreas from the *Wt* and *Reg4^-/-^* mice. Each group, n ≥ 7. Unpaired two-tailed Student’s *t* test with Welch’s correction analysis. **P* < 0.05.


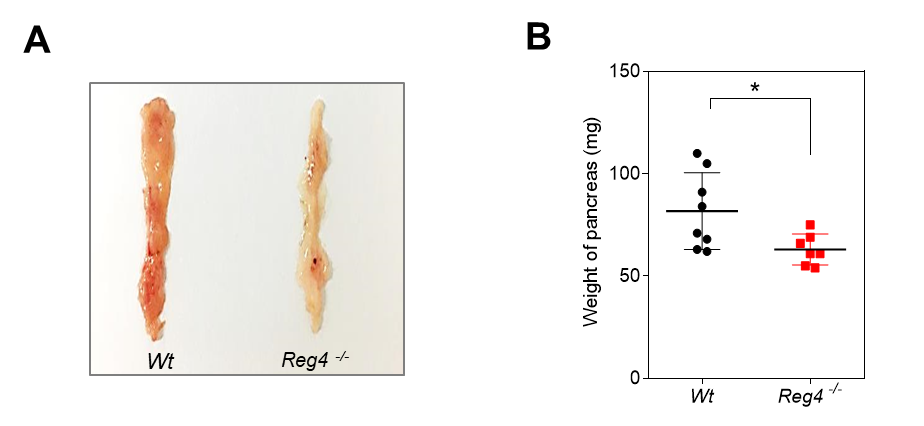


**Figure S4 Loss of *Reg4* leads to greater necrosis and inflammation in the pancreas during pancreatitis. A** Hematoxylin and eosin (H&E)-staining of pancreatic sections from *Reg4^-/-^* and *Wt* mice injected with caerulein and killed at 6 h, 24 h, 30 h, 2 weeks, 4 weeks, 6 weeks, or 8 weeks after the initial caerulein injection. **B** Quantification of the necrotic area as a percentage of the total tissue area on pancreatic sections (for each group, n ≥ 3). **C** Immune cell infiltrates during caerulein-induced pancreatitis, as visualized by immunohistochemical (IHC) staining against CD45. **D** Quantification of CD45-positive cells in each field (for each group, n ≥ 3); unpaired, two-tailed Student’s *t* test with or without Welch’s correction analysis for **B** and **D** (ns, not significant; **P* < 0.05, ***P* < 0.01, ****P* < 0.001).

**
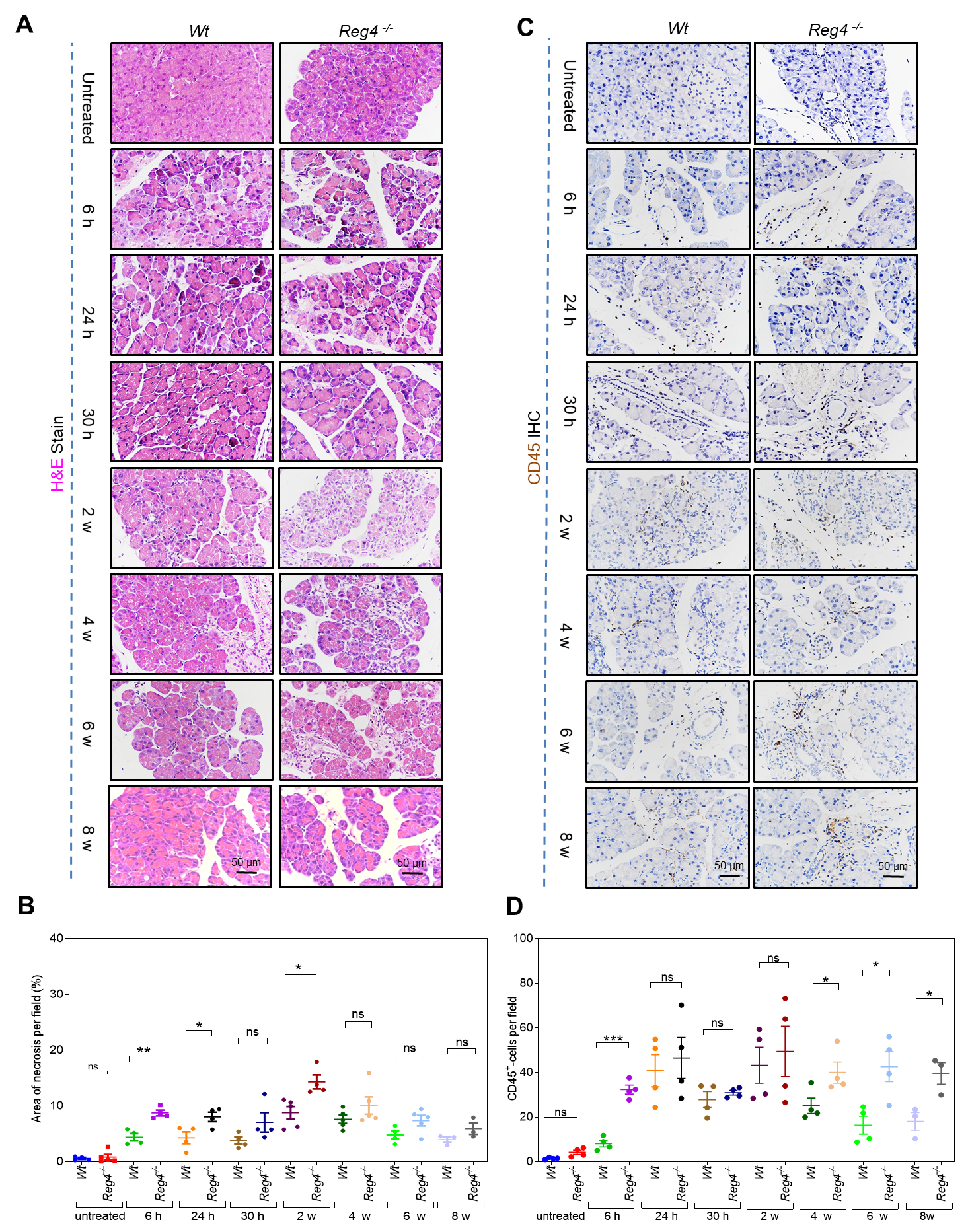
**

**Figure S5 *Reg4* loss alters macrophages infiltrates during the experimental pancreatitis. A** Immunofluorescence (IF) staining of pancreatic sections for CD45 (green) and F4/80 (red) during pancreatitis (each group n = 4, t = 24 hours, 2 weeks, 4 weeks, or 6 weeks). **B** Immunohistochemical (IHC) staining against F4/80 in pancreas of *Wt* and *Reg4^-/-^* mice (each group n = 4, t = 24 hours, 2 weeks, 4 weeks, or 6 weeks). **C** Quantification of the F4/80^+^ cells per field. Unpaired two-tailed Student’s *t* test with or without Welch’s correction analysis for **C**. ns, not significant, **P* < 0.05


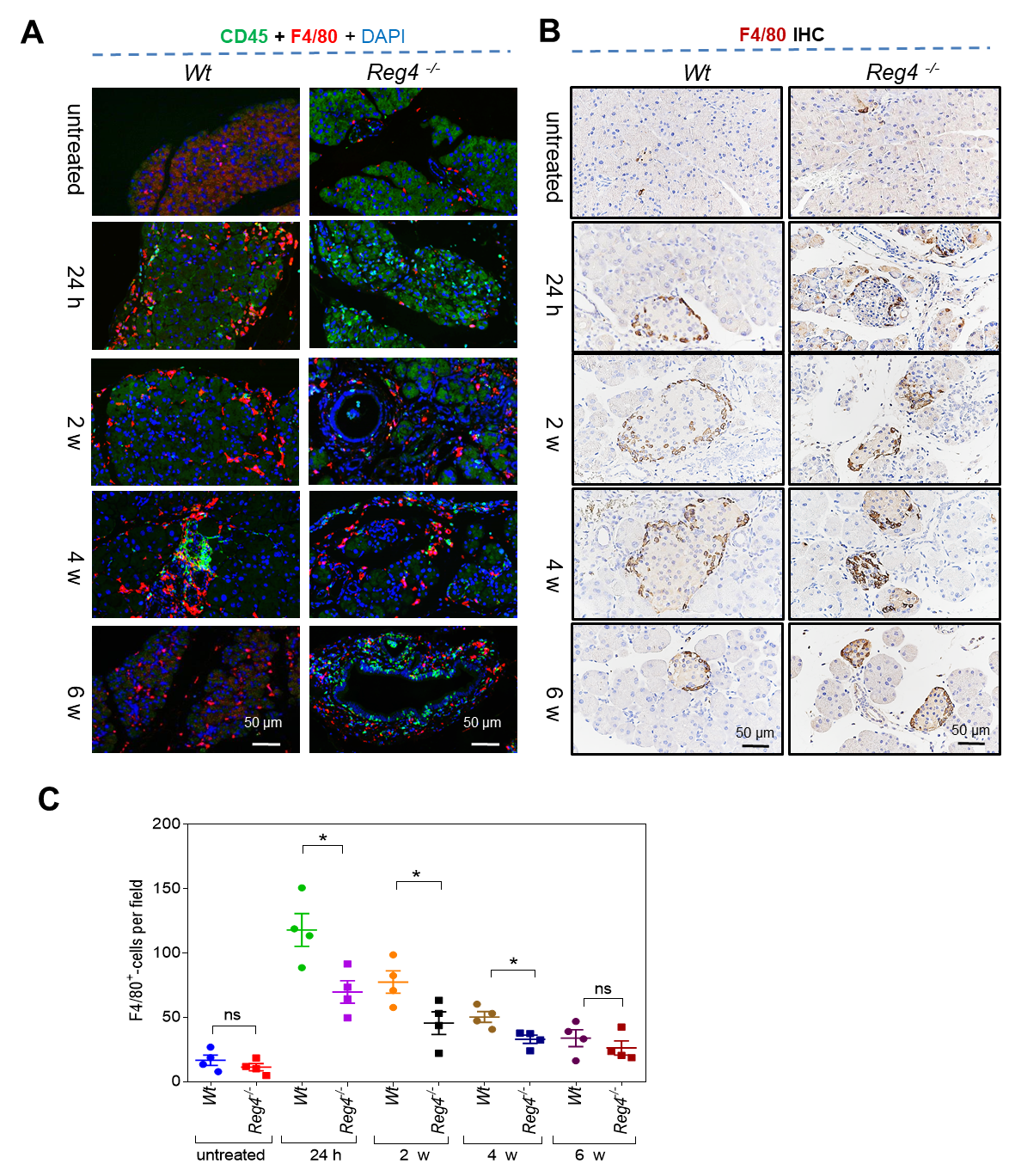


**Figure S6 *Reg4* loss reduces proliferation of pancreas during the experimental pancreatitis. A** Immunohistochemical (IHC) staining against Pcna of pancreatic sections from *Reg4^-/-^* and *Wt* mice injected with caerulein and killed 6 hours, 24 hours, 30 hours, 2 weeks, 4 weeks, 6 weeks or 8 weeks after the initial caerulein injection. **B** Quantification of the Pcna^+^ cell per field. Each group n ≥ 3. Unpaired two-tailed Student’s *t* test with or without Welch’s correction analysis for B. ns, not significant, **P* < 0.05, ***P* < 0.01, ****P* < 0.001


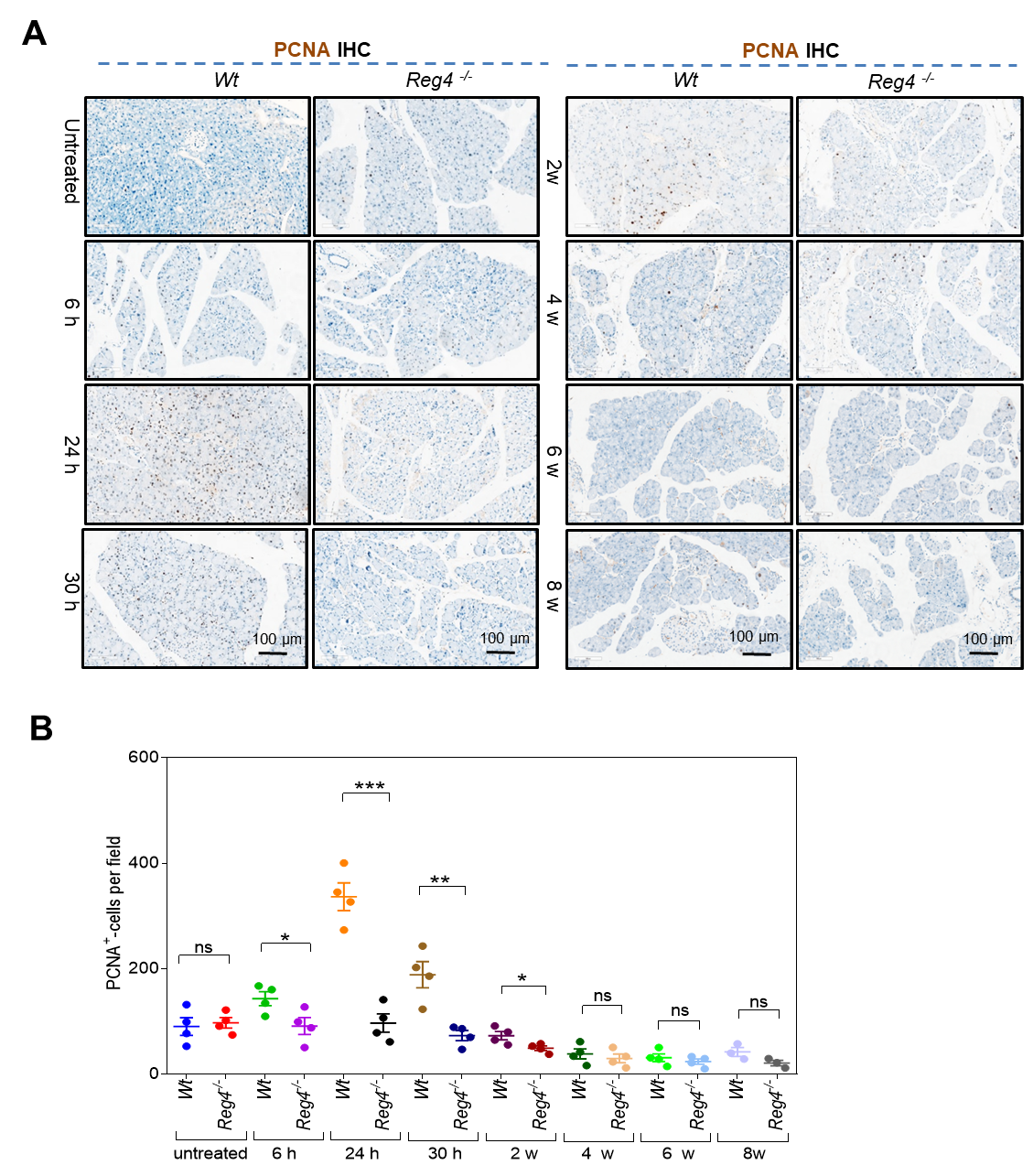


**Figure S7 *Reg4* deficiency decreases the Ki67-postive cells during the experimental pancreatitis. A** Immunofluorescence (IF) staining of pancreatic sections for Ki67 (green) during pancreatitis (each group n =4, t = 24 hours, 2 weeks, 4 weeks or 6 weeks). **B** Quantification of the Ki67^+^ cells per field. Unpaired two-tailed Student’s *t* test with or without Welch’s correction analysis for **B**. **P* < 0.05


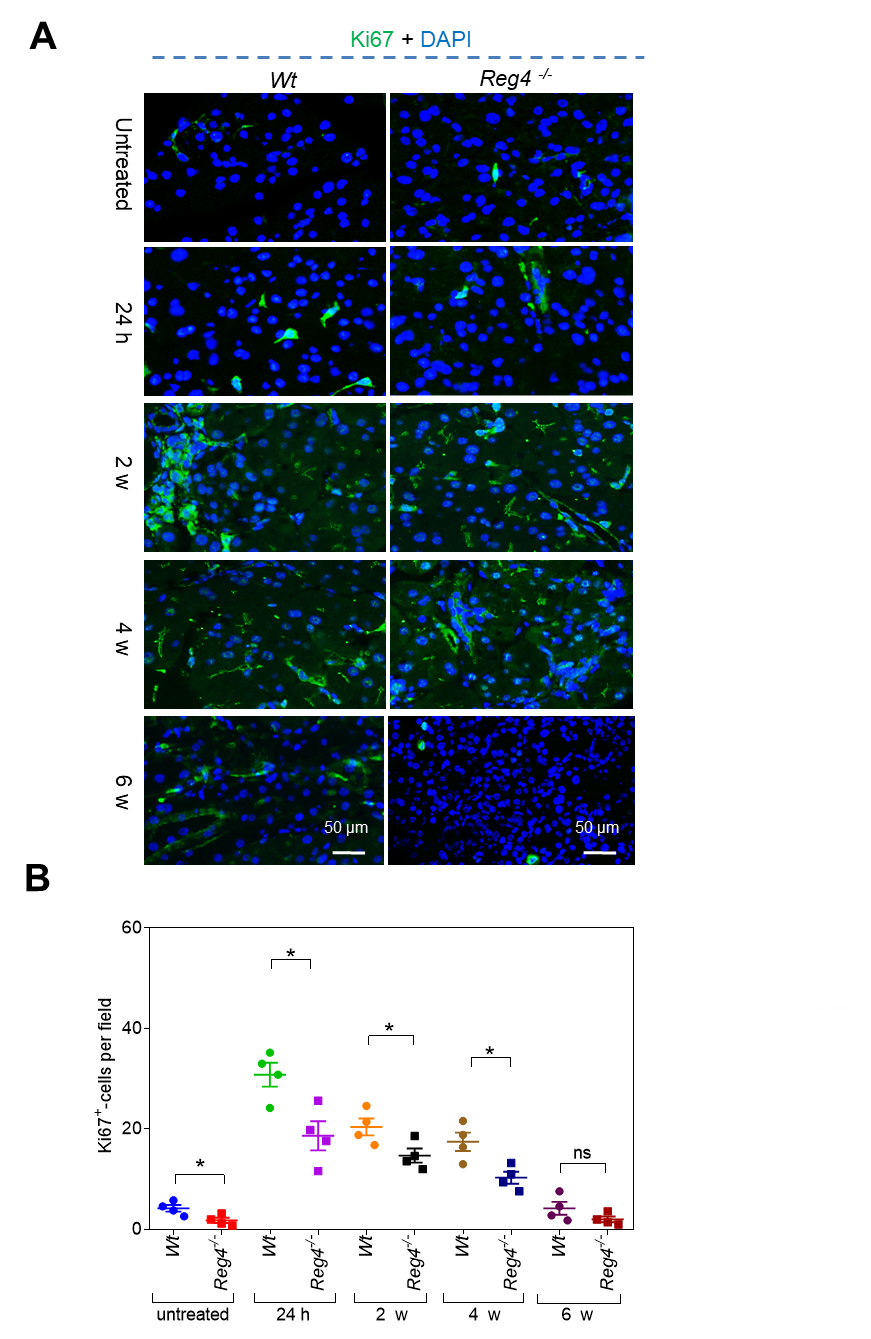


**Figure S8 *rReg4* administration ameliorates injuries in the pancreas during pancreatitis. A** Treatment schedule of mice during acute caerulein-induced pancreatitis with or without recombinant Reg4 protein (rReg4). *Reg4^-/-^* mice were treated with two injections (500 μg/kg body weight) started at 0 h and 8 h, and mice were killed at 24 h (n ≥ 6 for each group). **B** H&E staining of the pancreatic sections from *Wt* mice, *Reg4^-/-^*, and *Reg4^-/-^* administered rReg4; and left uninjected or injected with caerulein, and killed at 24 h. Quantification of the necrotic area as a percentage of the tissue area in panel B (n = 4 for each group). **C** Terminal deoxynucleotidyl transferase dUTP nick-end labeling (TUNEL) analysis of the pancreatic sections from *Wt* mice, *Reg4^-/-^*mice, and *Reg4^-/-^* mice with rReg4 administration. Quantification of the number of TUNEL-positive cells per field in panel C (n = 4 for each group). **D** Western-blot analysis for BCL-2, PUMA, cleaved caspase-3/9, caspase-3/9, and GAPDH in the pancreases of *Wt*, *Reg4^-/-^* mice, and *Reg4^-/-^* mice with rReg4 treatment (n = 4 for each group). **E** Quantification of panel D; GAPDH was used as the internal reference. Randomized one-way ANOVA for **B**, **C** and **E** (ns, not significant; **P* < 0.05, ***P* < 0.01, ****P* < 0.001).


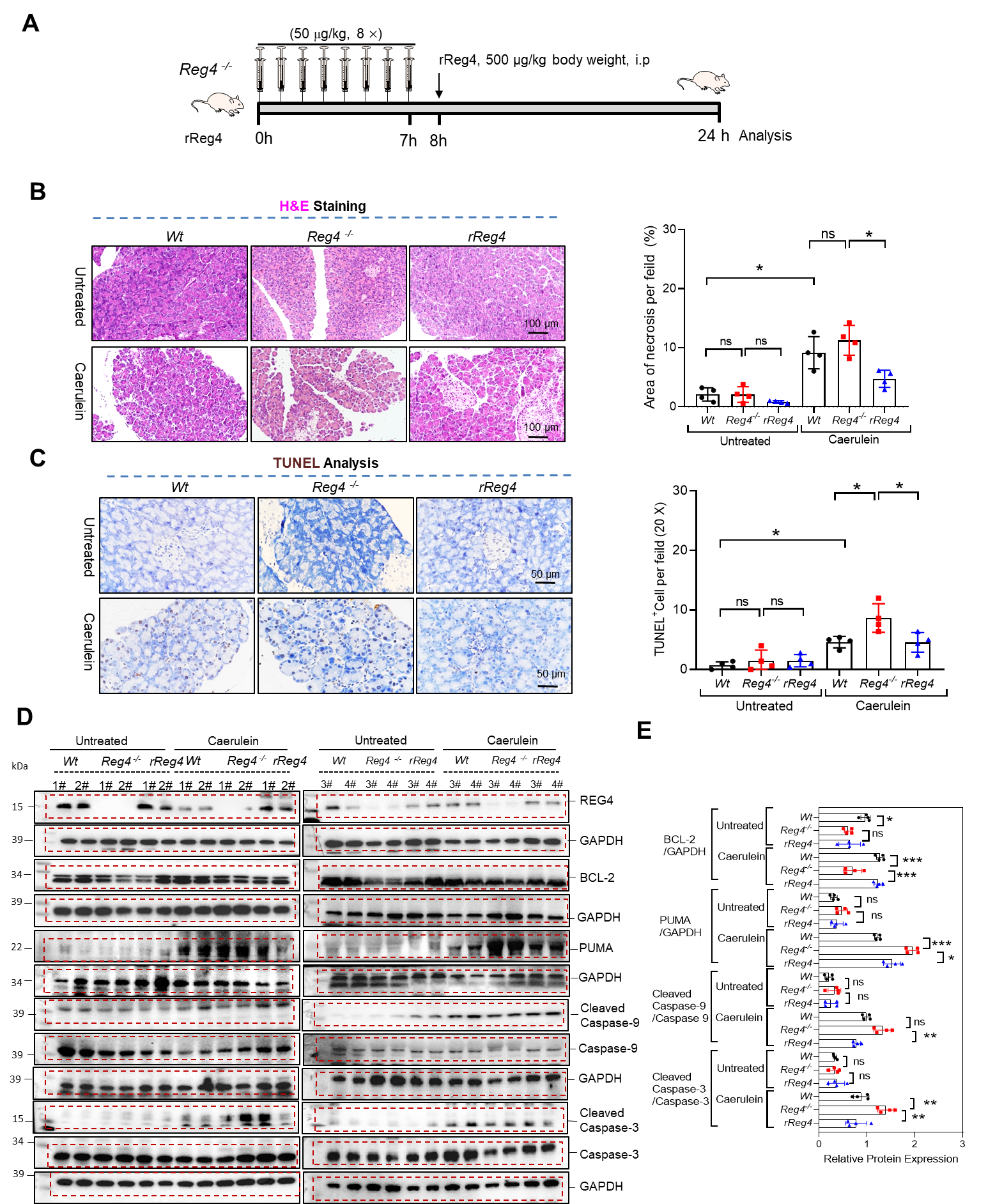


**Figure S9 Recombinant Reg4 protein administration protects the acinar cells from Caerulein- or Arginine-induced cell death. A** The acinar cells were isolated from the adult mice, and were treated with 200 μg/l Cerulein or 2 mg/ml arginine with or without 20 μg/ml recombinant Reg4 protein (rReg4), and lasted for 0, 2, 4, or 8 hours. Representative images of acinar cells. **B** Lactate dehydrogenase (LDH) cytotoxicity assay for the above treated acinar cells. Repeats n ≥3.


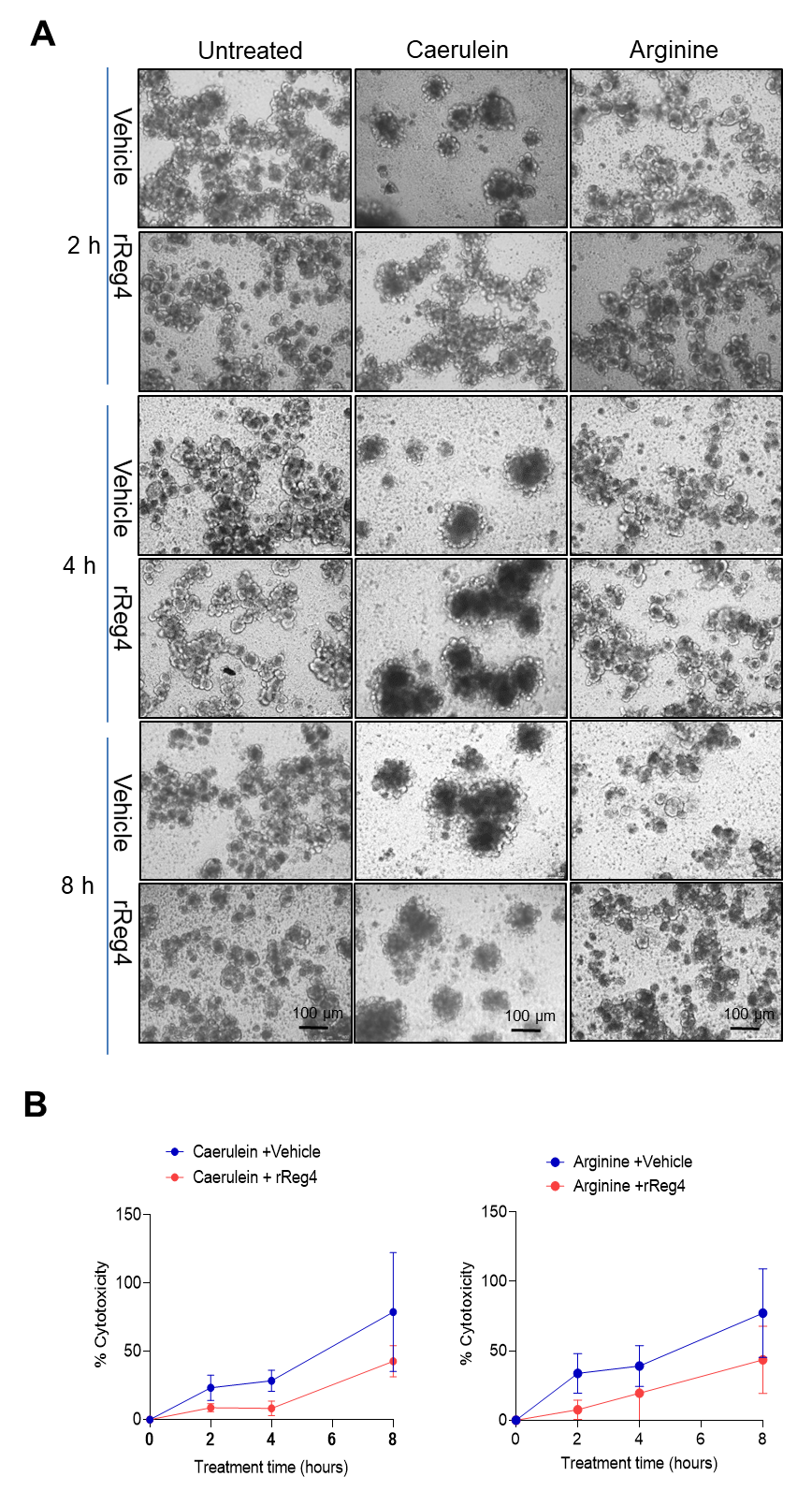


**Figure S10 rReg4 administration amolierates pancreatitis. A** Immunohistochemical (IHC) staining for CD45 in the pancreases of *Wt* mice, *Reg4^-/-^* mice, and *Reg4^-/-^* mice with rReg4 administration; and left uninjected or injected with caerulein, and killed at 24 h (n = 4 for each group). Quantification of the number of CD45-positive cells per field. **B** IHC staining for phosphorylated-STAT3 (p-STAT3) in the pancreas of *Wt* mice, *Reg4^-/-^*, and *Reg4^-/-^* with rReg4 administration (n = 4 for each group). Quantification of the number of p-Stat3-positive cells per field. **C** Western-blot analysis of CXCR4, CXCL12, and GAPDH in the pancreases of *Wt*, *Reg4^-/-^* mice, and *Reg4^-/-^* mice with rReg4 treatment (n = 4 for each group). **D** Quantification of panel C; Randomized one-way ANOVA for **A**, **B** and **D** (ns, not significant; **P* < 0.05, ****P* < 0.001).

**
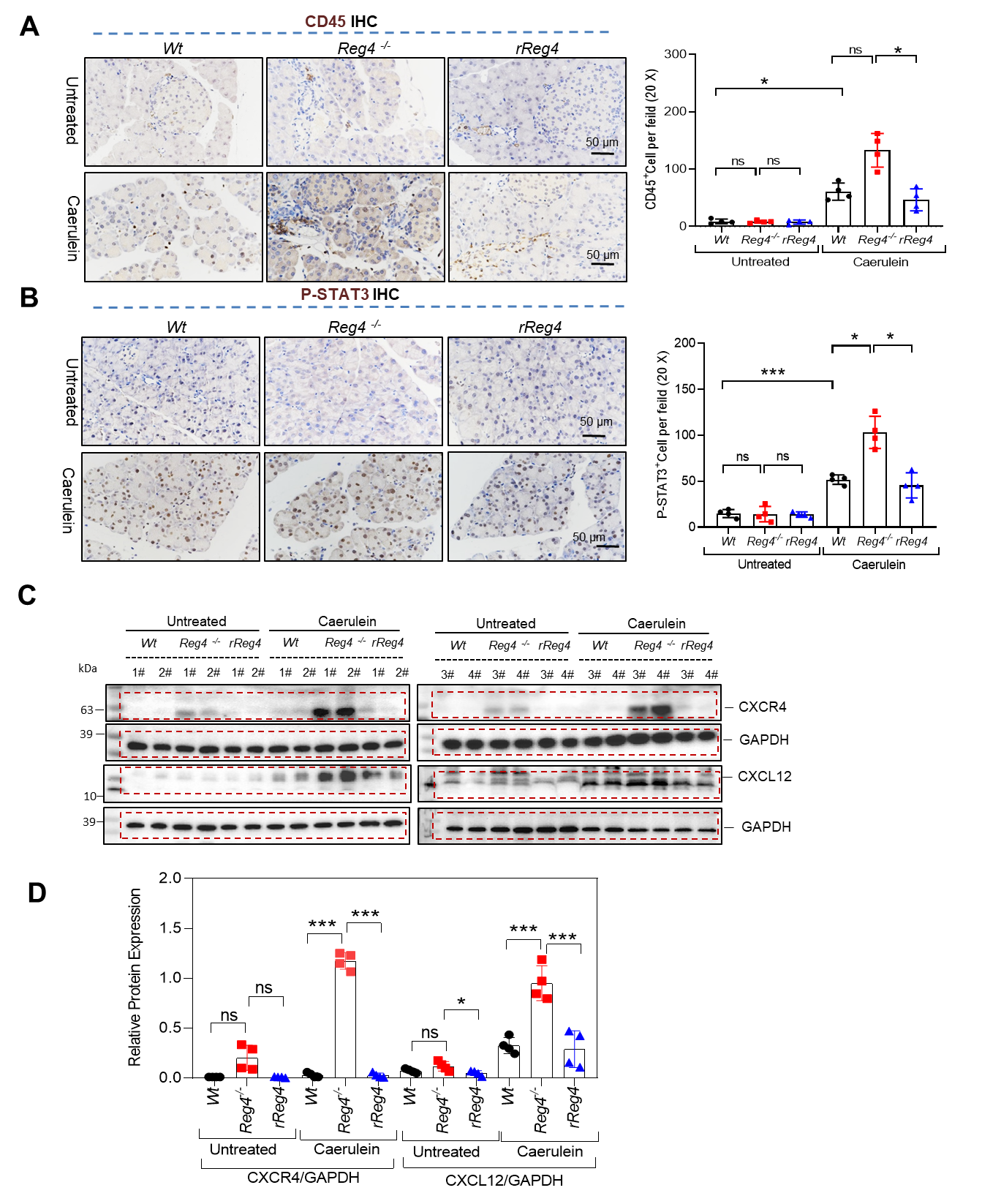
**
