# Supplementary material for: Reg4 deficiency aggravates pancreatitis by increasing mitochondrial cell death and fibrosis
Source: Cell Death Dis. 2024 May 20;15(5):348. doi: 10.1038/s41419-024-06738-y (PMC11106239; doi:10.1038/s41419-024-06738-y)
Supplement: Supplementary file 2 — Original WB bands [file 41419_2024_6738_MOESM2_ESM.docx]

# Supplementary Materials

***Reg4* deficiency aggravates pancreatitis by increasing mitochondrial cell death and fibrosis**

Weihui Yan, Ying Wang, Ying Lu, [Shicheng](https://www.ncbi.nlm.nih.gov/pubmed/?term=Chen%20S%5BAuthor%5D&cauthor=true&cauthor_uid=34168124) Peng, Bo Wu, Wei Cai and Yongtao Xiao

**Contents:**

Original WB bands in this study, Pages 2 - 13

**Original WB bands in this study**

Figure 5

**
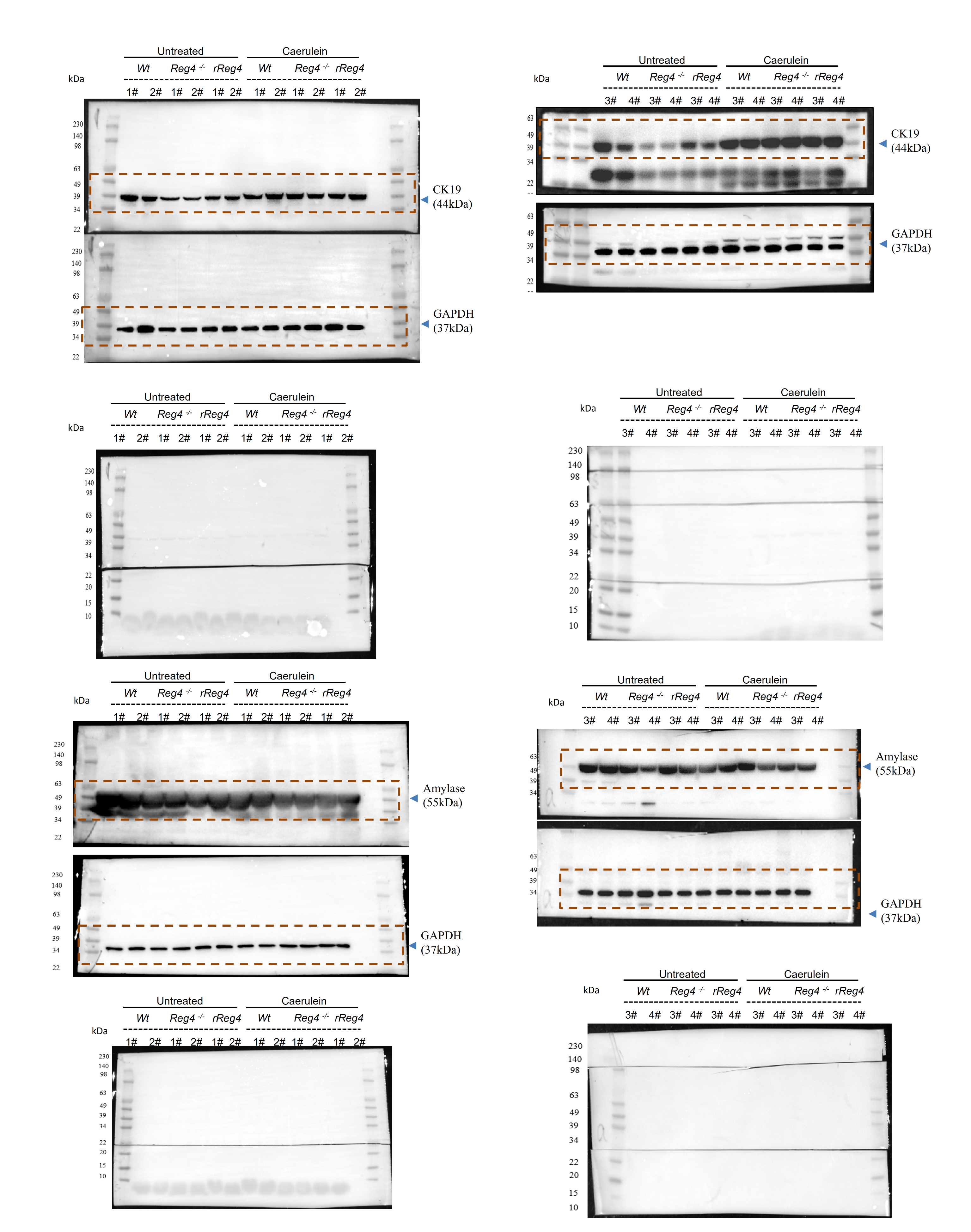
**

Figure 5

**
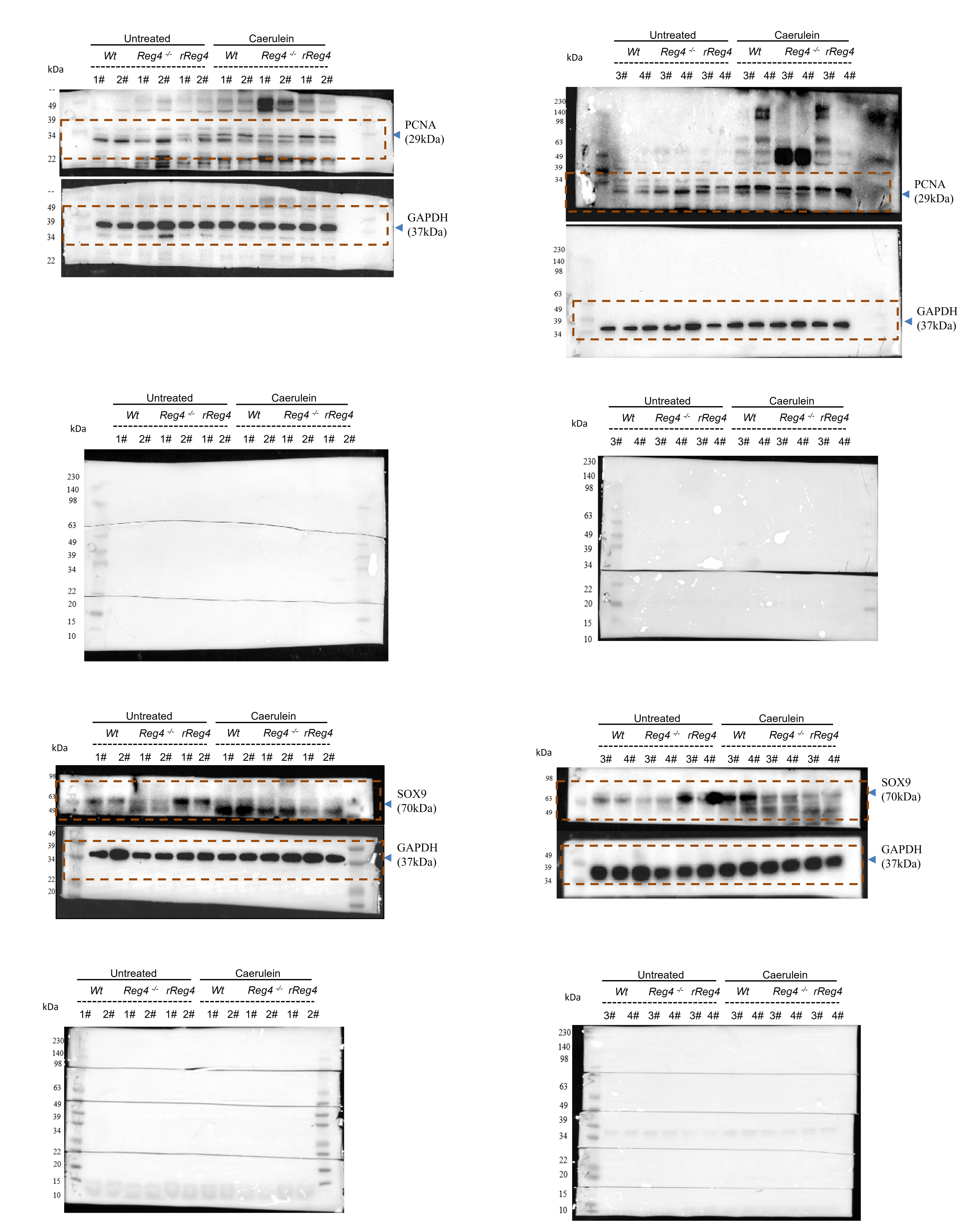
**

Figure 5


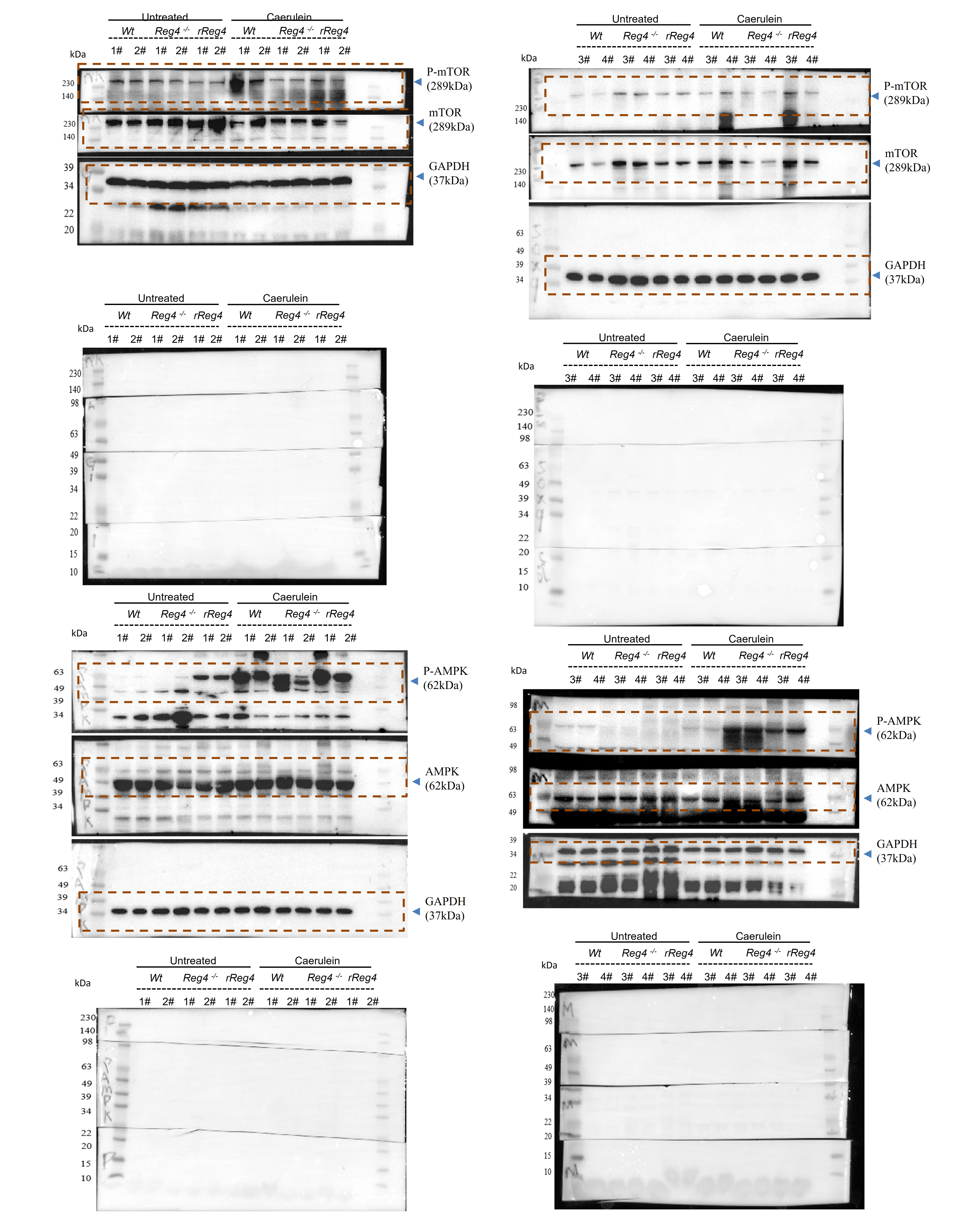


Figure 5


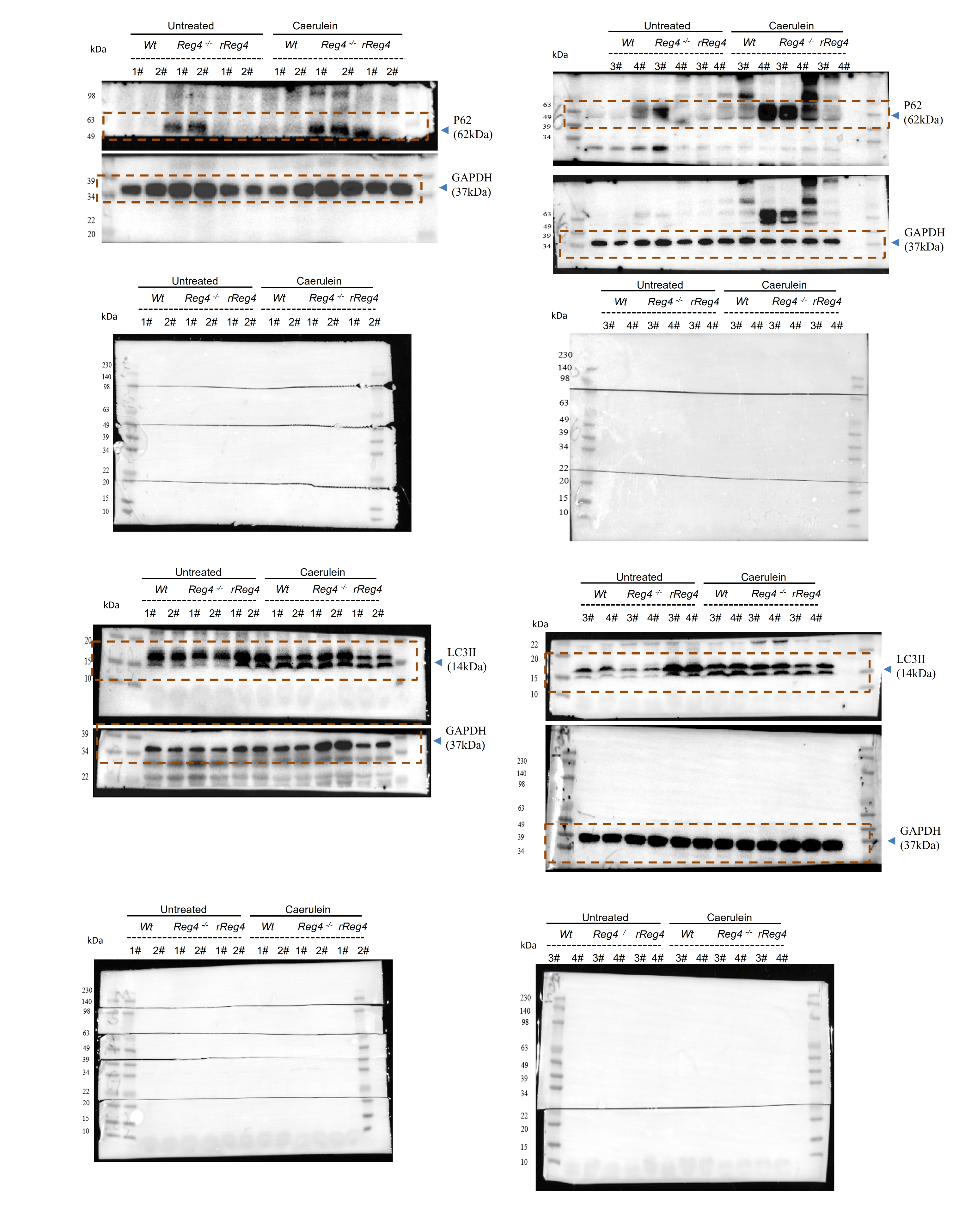


Figure 6


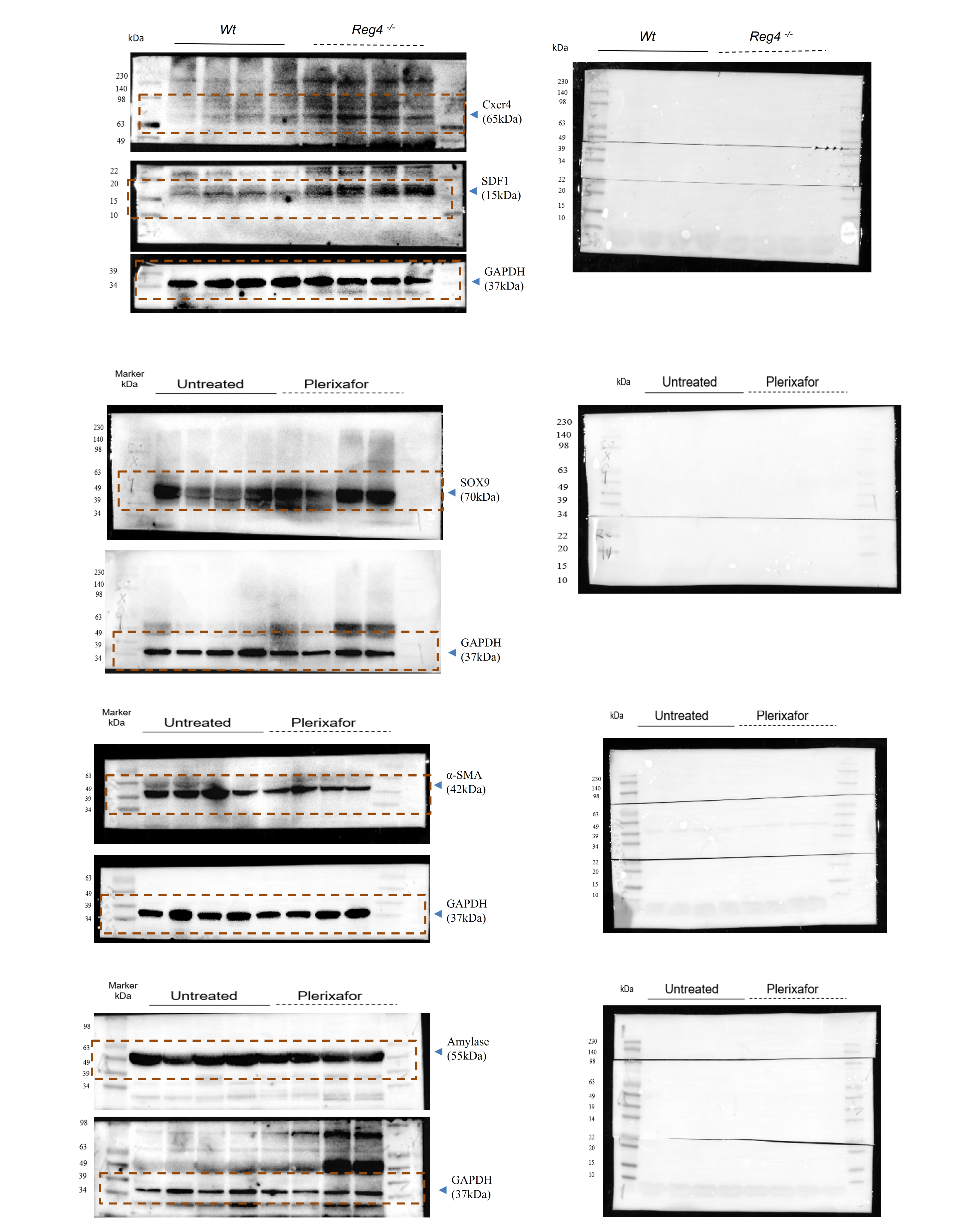


CXCL12

Figure 7


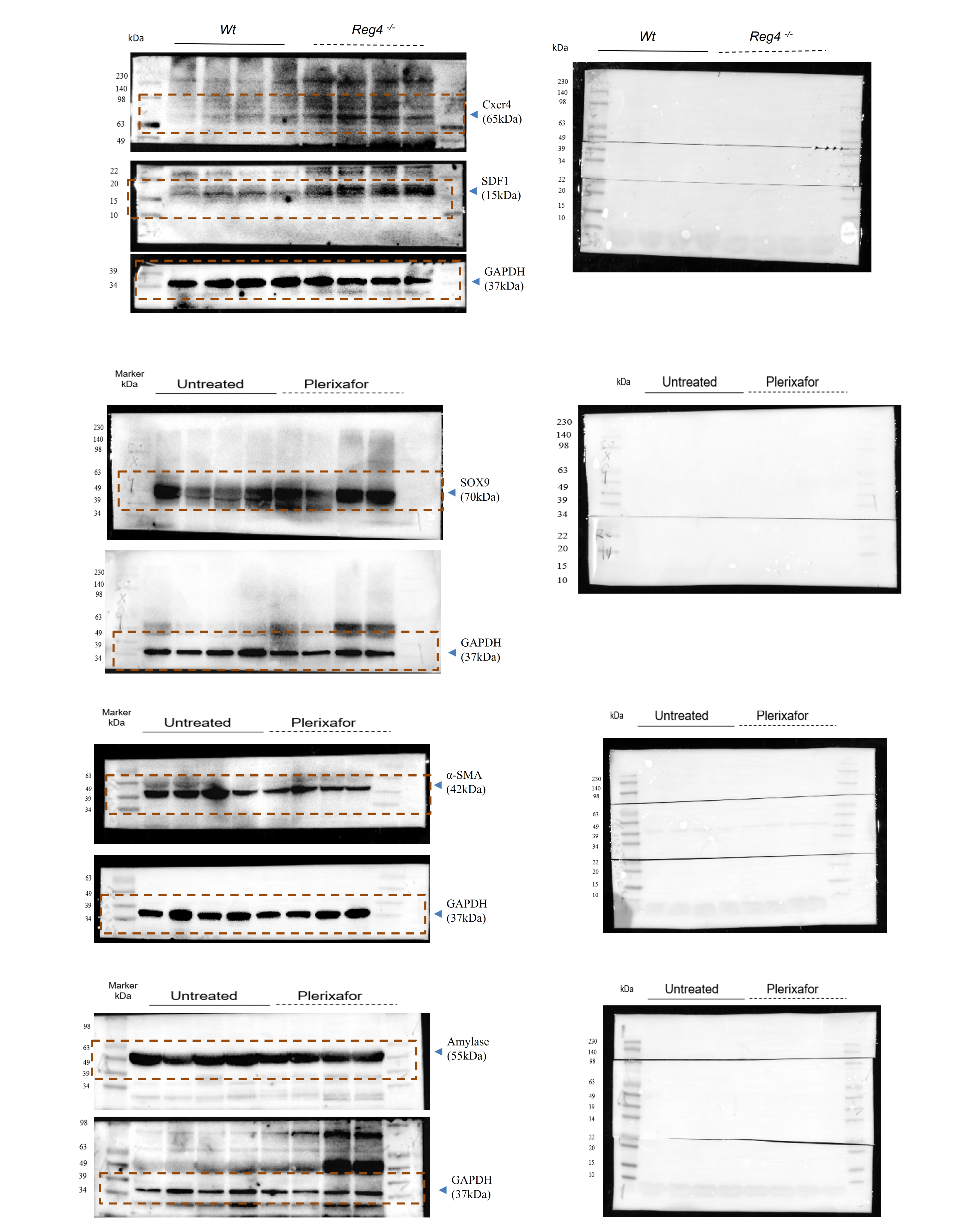


Figure 7


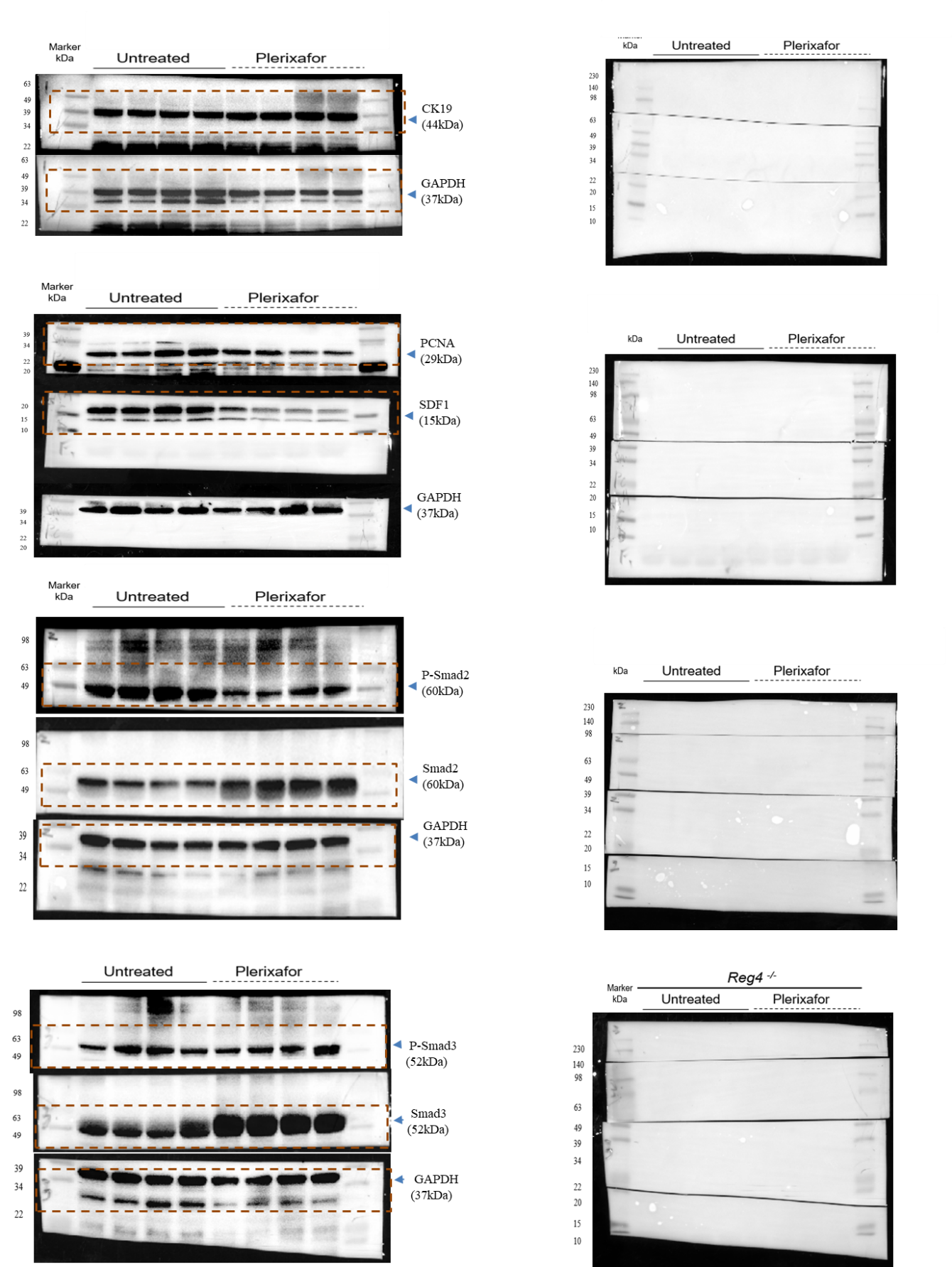


Figure 8


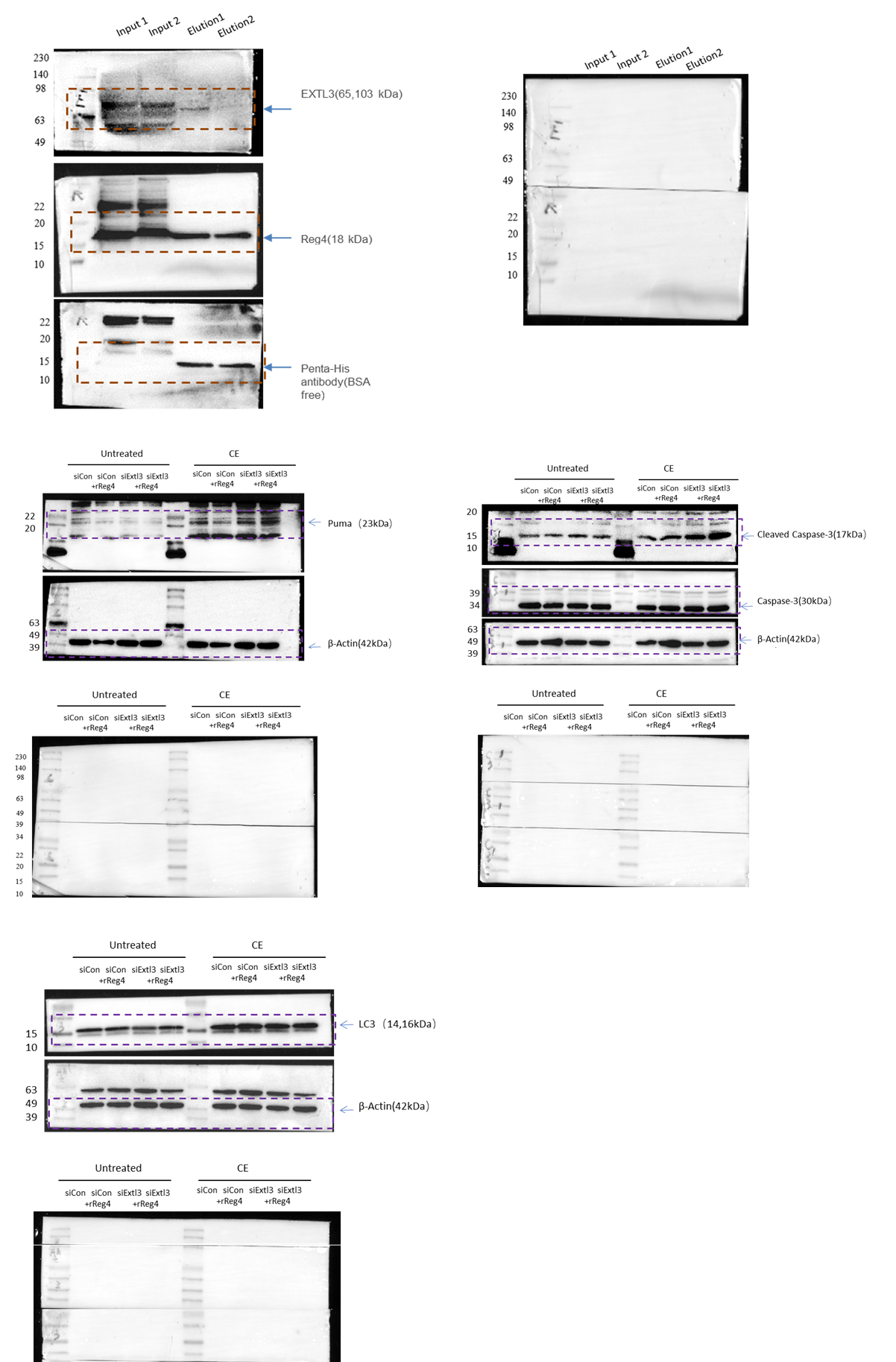


Figure S2


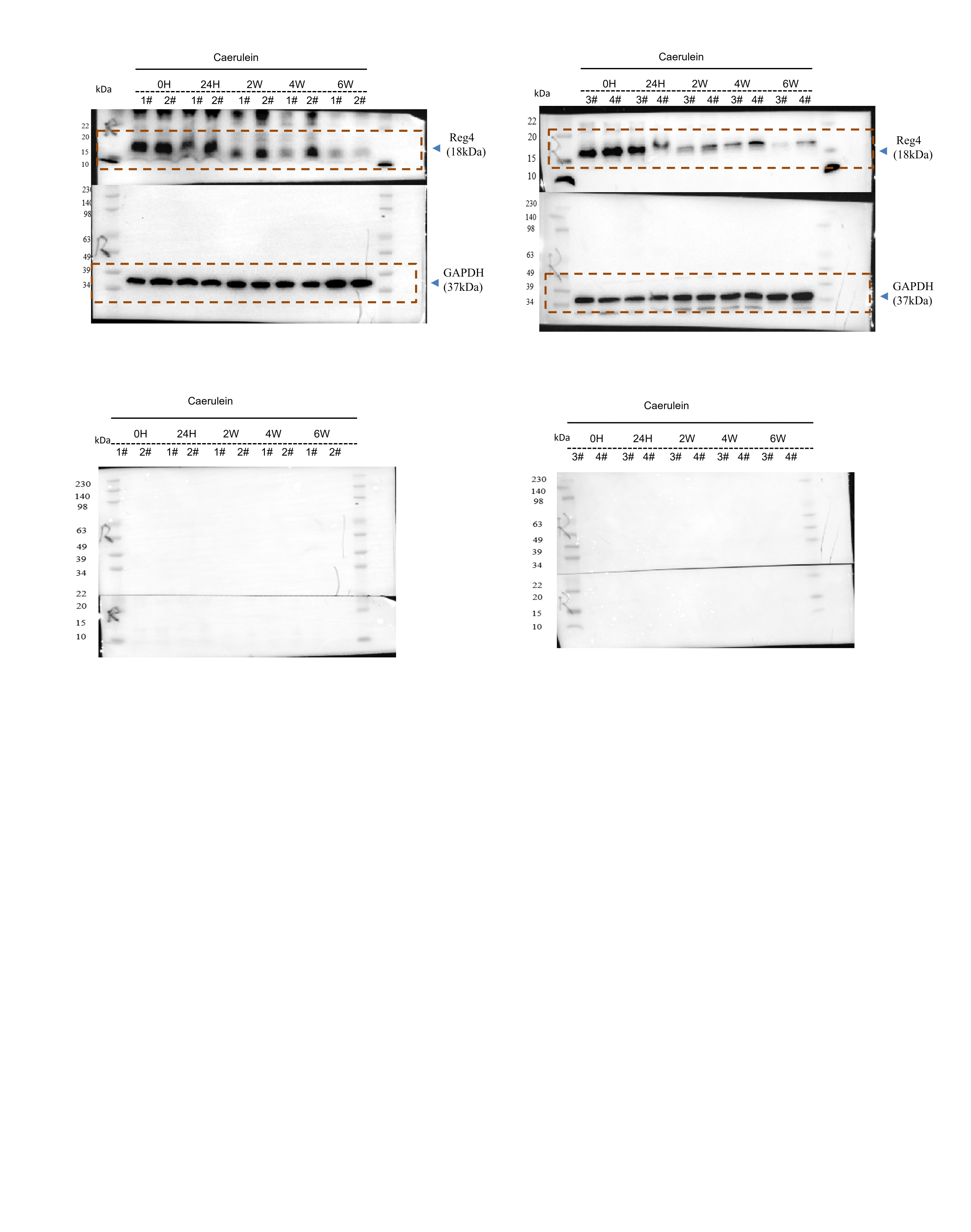


Figure S8

**
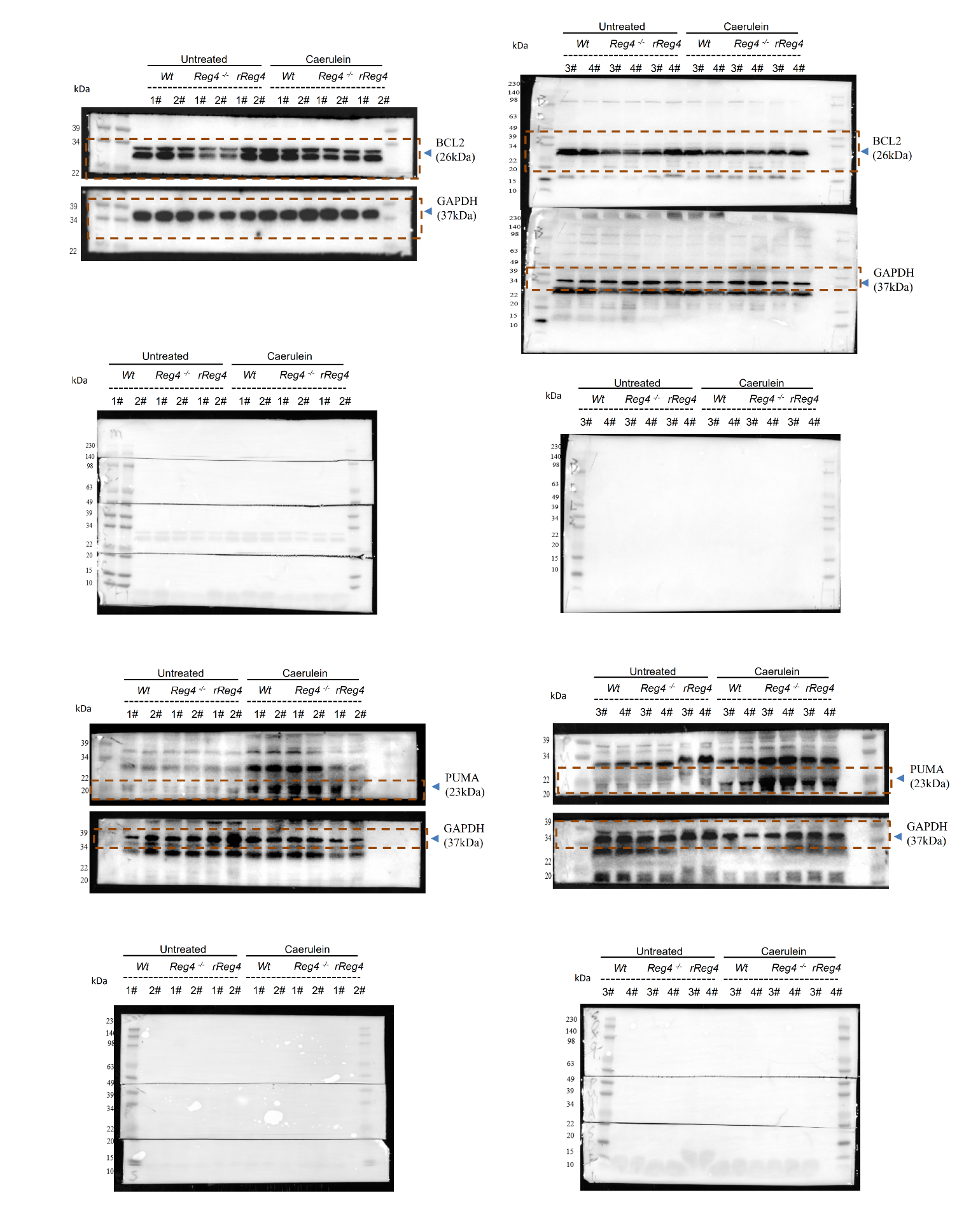
**

Figure S8

**
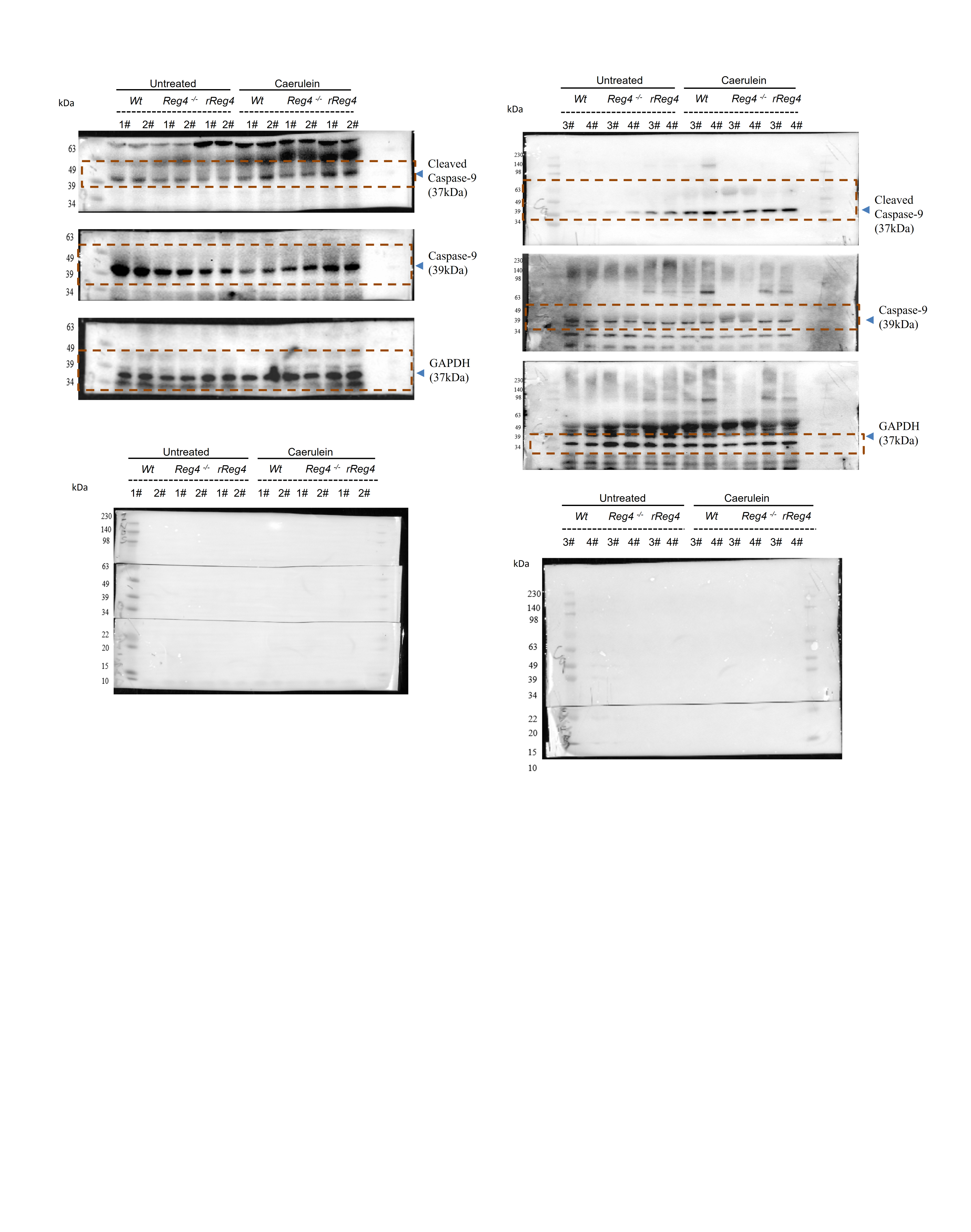
**

Figure S8

**
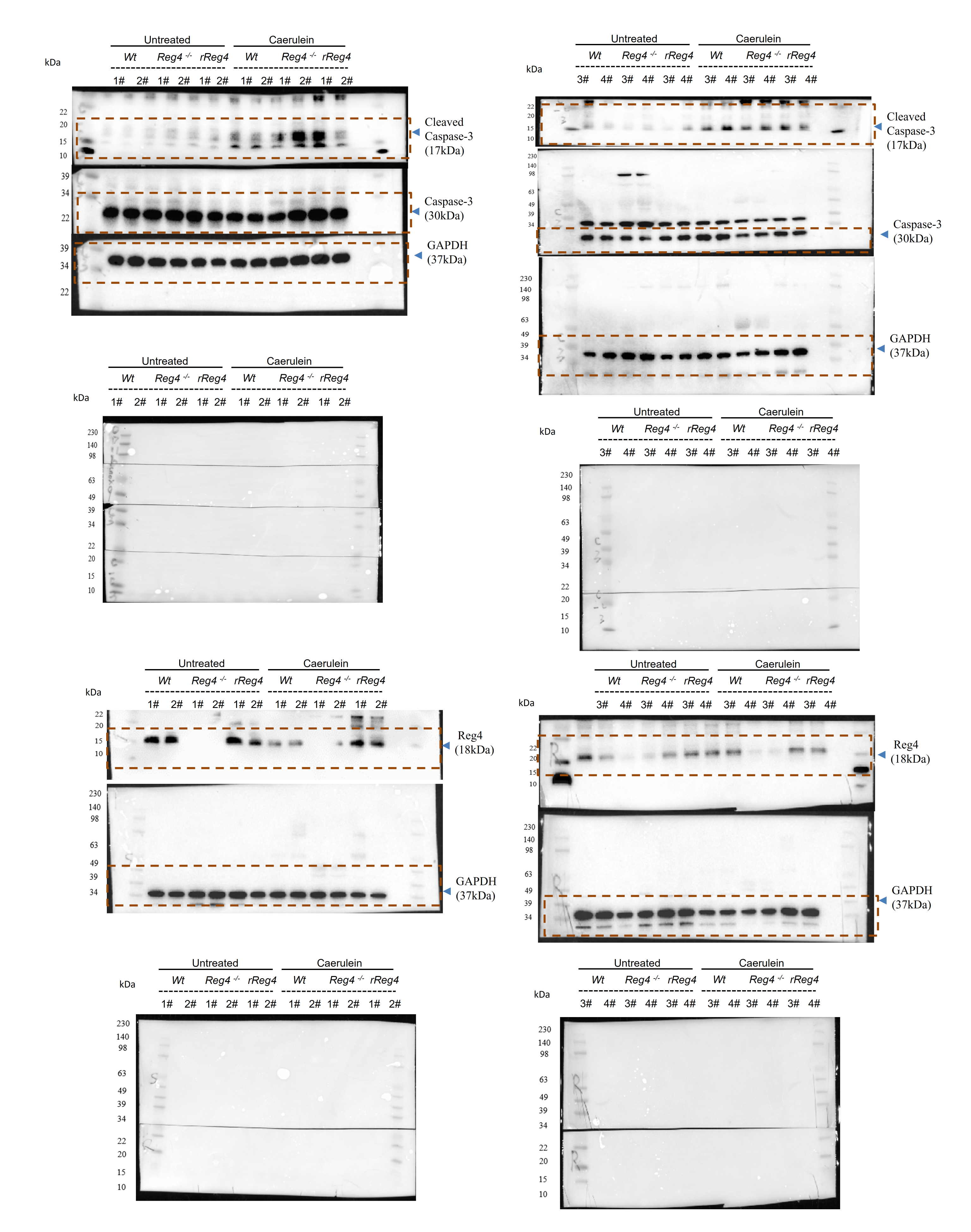
**

Figure S10

**
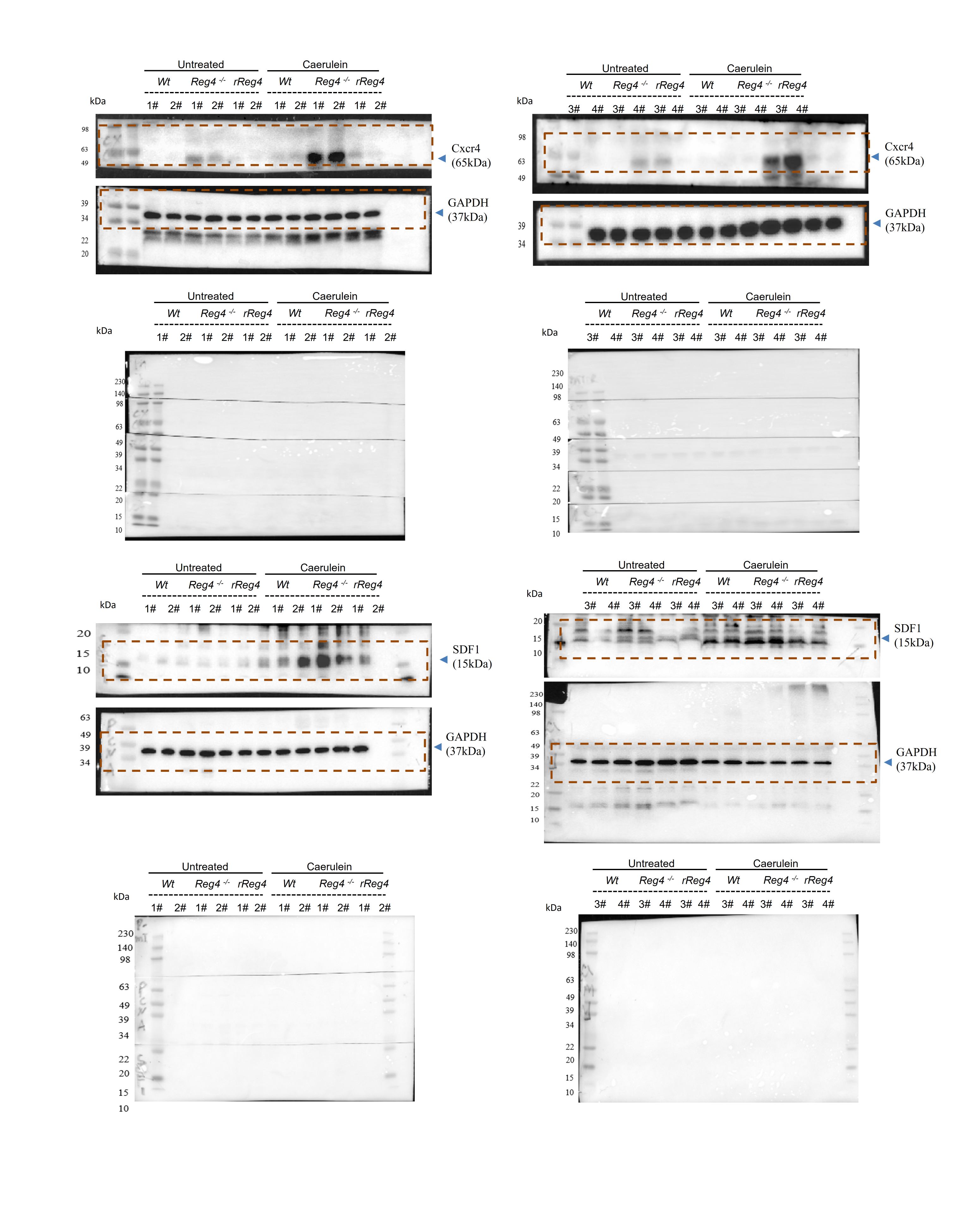
**
